# Evaluation of Shear Capacity of Steel Fiber Reinforced Concrete Beams without Stirrups Using Artificial Intelligence Models

**DOI:** 10.3390/ma15072407

**Published:** 2022-03-24

**Authors:** Yong Yu, Xin-Yu Zhao, Jin-Jun Xu, Shao-Chun Wang, Tian-Yu Xie

**Affiliations:** 1 School of Environment and Civil Engineering, Dongguan University of Technology, Dongguan 523808, China; yuyong1990@foxmail.com; 2 State Key Laboratory of Subtropical Building Science, South China University of Technology, Guangzhou 510640, China; 3 College of Civil Engineering, Nanjing Tech University, Nanjing 211816, China; jjxu_concrete@njtech.edu.cn; 4 Shanghai Construction No.1 (Group) Co., Ltd., Shanghai 200120, China; dazzlewood_wang@outlook.com; 5 School of Engineering, RMIT University, Melbourne, VIC 3000, Australia; xiety72@hotmail.com

**Keywords:** steel fiber reinforced concrete beam, shear capacity, back-propagation artificial neural work, random forest, multi-gene genetic programming, parameter sensitivity

## Abstract

The shear transfer mechanism of steel fiber reinforced concrete (SFRC) beams without stirrups is still not well understood. This is demonstrated herein by examining the accuracy of typical empirical formulas for 488 SFRC beam test records compiled from the literature. To steer clear of these cognitive limitations, this study turned to artificial intelligence (AI) models. A gray relational analysis (GRA) was first conducted to evaluate the importance of different parameters for the problem at hand. The outcomes indicate that the shear capacity depends heavily on the material properties of concrete, the amount of longitudinal reinforcement, the attributes of steel fibers, and the geometrical and loading characteristics of SFRC beams. After this, AI models, including back-propagation artificial neural network, random forest and multi-gene genetic programming, were developed to capture the shear strength of SFRC beams without stirrups. The findings unequivocally show that the AI models predict the shear strength more accurately than do the empirical formulas. A parametric analysis was performed using the established AI model to investigate the effects of the main influential factors (determined by GRA) on the shear capacity. Overall, this paper provides an accurate, instantaneous and meaningful approach for evaluating the shear capacity of SFRC beams containing no stirrups.

## 1. Introduction

Concrete is currently the most widely used construction material [1,2]. However, it has some fundamental weaknesses: low tensile capacity and increased brittleness with strength. Engineers considered adding ductile steel fibers to plain concrete to overcome this drawback, and a new composite—steel fiber reinforced concrete (SFRC)—was thus formed [3,4]. This treatment is simple to implement and has a beneficial effect on improving concrete’s crack-resisting capacity [5]. Additionally, the durability of concrete can be substantially improved, because cracking is well controlled. From a long-term point of view, SFRC can also be deemed environmentally friendly since it indirectly reduces concrete consumption as a result of the increased service life of SFRC components [6].

As early as 1874, Bernard proposed a method of reinforcing concrete with steel splinters, which is recognized as the earliest exploration of using steel fibers in concrete. Since then, SFRC has received immense attention. Many studies have been conducted to investigate the mechanical properties [7,8,9,10], durability [3,11,12,13,14,15], fatigue performance [16,17] and microstructure characteristics of SFRC [18,19,20]. Based on these research results, it has been determined that SFRC is structurally superior to its ordinary concrete counterpart, especially under tension, shear or torsion forces [4]. Fibers can effectively prevent crack opening and carry further tension stress via bridging effects [8,9]. SFRC also experiences much less creep and shrinkage than plain concrete, owing to the improved fine microstructure in combination with reduced micro-cracks when adding fibers [21]. All of these factors explain the effectiveness and potential of SFRC.

However, it should be noted that the practical use of SFRC has lagged behind its scientific research [22]. Presently, its use is mainly limited to low-rise industrial structures, small housing buildings and some special locations (e.g., joints) in structures [23]. The reasons behind this are multifold [24]. One is that there is a lack of reliable models that can accurately elucidate the load-resisting mechanisms of members made with SFRC, especially for SFRC beams without stirrups [25]. As a matter of fact, in practice, it is desirable to use steel fibers in beams in order to reduce the labor-intensive work involved in making steel stirrups. This is particularly true for large girders. However, even with the addition of steel fibers, SFRC beams without stirrups tend to be more brittle in shear than SFRC beams with stirrups, and the quantity of fibers that should be included in a beam so that the fibers act as shear reinforcement instead of using stirrups is still inconclusive. This issue has attracted the attention of many experts aiming to reveal the mechanism behind it. Slater et al. [26], for example, compared the experimentally obtained shear strengths of 222 beams with those predicted using design equations. The predictions were not satisfactory, since these equations did not fully consider the influential parameters involved. Voo et al. [27] questioned the assumption that steel fibers only contribute to the shear-resisting mechanism across a crack, as they observed a clear reduction in the deflection of SFRC beams. Recently, Lantsoght et al. [22,28] indicated that steel fibers affect all five shear transfer mechanisms in ordinary concrete beams (in this study, “ordinary” means a beam without fibers), and these alterations pose a significant challenge to the shear design of SFRC beams without stirrups.

To exploit the benefits of using steel fibers, optimize component design and ensure structural safety, it is necessary to develop accurate and practical models for determining the shear bearing capacity of SFRC beams containing no stirrups.

Over the past two decades, artificial intelligence (AI) models have gained much attention due to the accuracy, simplicity and reliability of these data-driven approaches. As a result, they have been gradually accepted by the civil engineering community for predicting different properties of materials and structures [29]. AI models often outperform mechanics-based models, as the former can exploit hidden patterns between seemingly unrelated parameters to draw useful solution(s) [30,31].

Scholars have also proposed a variety of AI-based approaches to investigate the shear capacity of SFRC beams [32,33,34]. For example, Keshtegar et al. [32] used a response surface method–support vector regression hybrid method (RSM–SVR) to address this issue. They observed that their method had superior accuracy, outperforming the individual RSM or SVR method. Rahman and his co-workers [33] used a total of 11 models to predict the SFRC beam’s shear strength and found that the XGBoost algorithm had the highest precision. Chaabene and Nehdi [34] adopted a genetic programming-based symbolic regression (GP-SR) method to address the same topic. Unlike the two previous studies, this work proposed a novel tabular generative adversarial network (TGAN) technique that generates 2000 synthetic data points to address the lack of experimental data.

These pioneering investigations are of great significance for improving the shear strength evaluation of SFRC beams. However, generally speaking, there are still some shortcomings, mainly: (i) Not all of the decisive factors were considered or well-reflected in the previous models. For example, neither the effect of beam sectional width nor the effective depth was considered in [33]. (ii) The AI-based models do not usually provide an explicit formula. They resemble a black box, which has attracted some criticism.

Facing the above concerns, the main objective of this study is to develop automated approaches with higher accuracy by taking more comprehensive parameters into account. To achieve this goal:

- First, the influence of using steel fibers on the SFRC’s basic mechanical properties and the reinforced SFRC beam’s shear bearing capacity are briefly reviewed. In addition, the database of SFRC beams containing no stirrups established by Lantsoght [28] is succinctly analyzed.
- A gray relational analysis is then performed to identify the parameter importance.
- AI models, including back-propagation artificial neural network (BPANN), random forest (RF) and multi-gene genetic programming (MGGP), are developed to simulate the shear strength of the reinforced SFRC beam without stirrups.
- A parametric study is finally carried out to validate and explain the AI models.

Through the above steps, this study demonstrates how AI models can be introduced as a contemporary tool to augment the shear capacity evaluation of SFRC beams containing no stirrups.

## 2. Literature Review, Experimental Database and Typical Prediction Models

### 2.1. Literature Review

Dispersing steel fibers in concrete is beneficial for improving the mechanical properties of concrete. A review of the experimental results reported in the literature [3,4,5,6,7,8,9,10] indicates that: (a) Adding steel fibers is conducive to increasing concrete’s compressive strength, but the improvement effect is usually small compared with that for tension-related properties. Generally, the optimum dosage of steel fibers is a volume fraction of about 1.0%, which leads to an increase in the compressive strength of 8.1–14.0%. (b) As the amount of steel fibers increases, the SFRC’s elastic modulus increases moderately, and this can be well predicted using a series model. (c) Adding steel fibers can significantly increase the tensile strength and post-peak ductility. In general, 1.0% volume content of steel fibers could increase the SFRC’s splitting tensile strength by 15–40%. Additionally, with an increase in the fiber content, the tensile failure pattern progressively evolves from single-cracking to multi-cracking behavior. (d) The mechanical properties of concrete are likely to deteriorate when the steel fiber content is greater than 2.0%, which is mainly caused by the increase in the heterogeneity of the concrete mixture.

Once under shear, steel fibers also play a key role due to the stress transfer between crack faces. This study focuses mainly on the effect of using steel fibers on the shear capacity of SFRC beams containing no stirrups. The research results reported by Singh and Jain [23], Manju et al. [24], Ashour et al. [25], Pansuk et al. [35], Kim et al. [36] and Zhao et al. [37] have shown that the shear strength of this kind of beam can increase by 14.9–72.9% relative to ordinary beams when the fiber dosage is 1.5%.

Regarding ordinary beams under shear loading, it has been accepted that there exist five common shear-resisting mechanisms, namely, concrete crushing, concrete cracking, aggregate interlock, dowel action and arch action (for members with a small shear span-to-depth ratio). When it comes to SFRC beams, Lantsoght [22] stated that these mechanisms change significantly: (a) Compared with ordinary beams, the compression zone in an SFRC beam is deeper as a result of a change in the horizontal equilibrium in the cross-section: the compression demand is thus higher, caused by more concrete participates in tension. (b) Naturally, using steel fibers significantly increases the resulting tensile force of concrete. (c) Aggregate interlock action for the SFRC material is also enhanced (though not very clearly understood). (d) Dowel action is the shear contribution of flexural reinforcement. The maximum resistance that can be developed through dowel action is related to the tensile strength of the concrete cover, which fails through splitting when the dowel action force becomes too large. Since both the tensile strength of SFRC and the concrete–reinforcement interfacial bond are improved, dowel action consequently becomes stronger in SFRC beams. (e) The addition of steel fibers could improve the arching action in SFRC (requires further study), as steel fibers help sustain the shear transferred across the critical crack in the compression strut, and the softening effect of the diagonal concrete strut weakens.

### 2.2. Experimental Database for Shear Testing of SFRC Beams without Stirrups

The database of SFRC beams containing no stirrups used herein was compiled by Lantsoght [28] from 63 independent works (see the supplementary data of this paper for details, Supplementary Materials). The whole database comprises 488 tests of SFRC beams containing longitudinal rebar (mild steel only) and no shear reinforcement (screening rules to determine usable shear test results are detailed in [28]; they commonly include casting and curing conditions and loading and measuring methods). Almost all of the tests reported were carried out on simply supported beams under three- or four-point bending, with two exceptions: (i) two-span beams in [38] that generated a negative moment at the middle support and (ii) six short-span beams in [39] that used special setups to prevent the development of arching action.

As presented in the supplementary data, each dataset includes the following information: the geometry and loading conditions of beams (*b* is the sectional width, *h* denotes the sectional depth, *d* represents the sectional effective depth, and *a*/*d* indicates the shear span-to-effective depth ratio), the properties of concrete (*f*_c_ is the SFRC’s compressive strength, and *s*_max_ is the maximum dimension of coarse aggregate), the area ratio of longitudinal tensile reinforcement (*ρ*_s_) and the attributes and amounts of steel fibers (including fiber type, fiber tensile strength *f*_f_, fiber diameter *d*_f_, fiber aspect ratio *l*_f_/*d*_f_ and fiber volume fraction *V*_f_). Each specimen’s ultimate shear strength is denoted as *V*_u_. Note that the parameter *ρ*_f_ is also included in the table and is used to represent the bond condition between the steel fiber and concrete. The value of *ρ*_f_ is 1.00 for hooked fibers, 0.75 for crimped fibers and 0.50 for straight fibers, as suggested in [30].

The most influential variables that control the shear strength (stress) *v*_u_ (defined as *V*_u_/*bd*) should be identified first. Previous research [40] on ordinary concrete beams has suggested that both concrete and longitudinal bars play a key role in resisting applied shear force. To describe these contributions, the following parameter selection scheme was adopted: (i) the parameters *f*_c_ and *s*_max_ were selected to quantify the contributions of concrete and aggregate interlock action, and (ii) the parameter *ρ*_s_ was employed to reflect the shear contribution of the longitudinal bar. The reinforcement index *F*, defined as *V*_f_*ρ*_f_*l*_f_/*d*_f_ [22], was used to characterize the internal confining effect of steel fibers. Furthermore, several other parameters, including *d*/*b* and *a*/*d*, were used in subsequent analyses, mainly to incorporate the effects of beam geometry and loading type. The above-mentioned parameters have been well documented for each collected specimen.

Table 1 reports the ranges and statistics of the parameters *d*/*b*, *a*/*d*, *f*_c_, *s*_max_, *ρ*_s_ and *F*, together with the shear strength *v*_u_, for all 488 specimens. It is clear that the database contains both slender and deep beams, and most of the specimens were manufactured with normal-strength concrete. In addition, for SFRC beams, the volume fraction and geometric length of steel fibers were mostly distributed in the ranges of 0–1.5% and 25–60 mm, respectively. The most frequently used shapes of steel fibers were hooked (63% of all compiled beams), crimped (22%) and straight smooth (3%).

### 2.3. Assessing Existing Prediction Models for Shear Capacity of SFRC Beams without Stirrups

**Figure 1:**
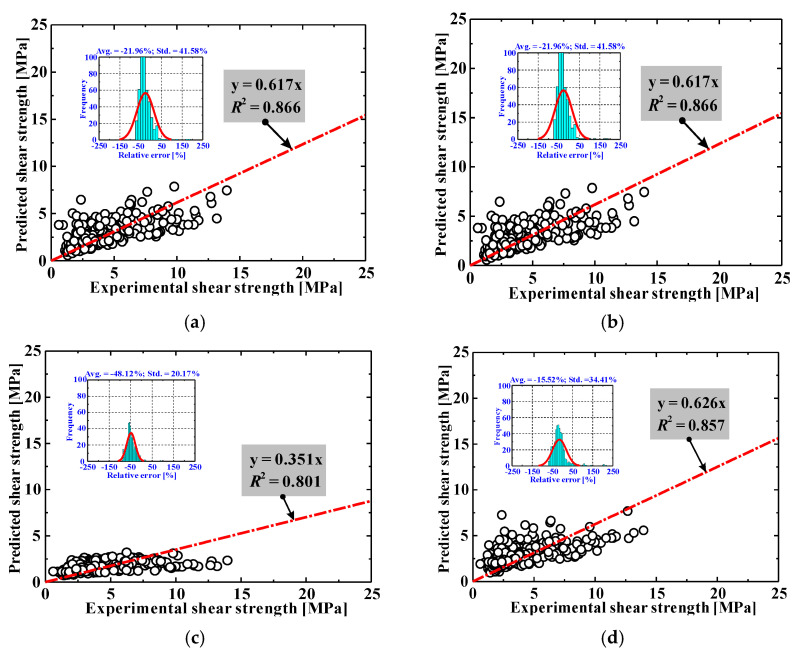

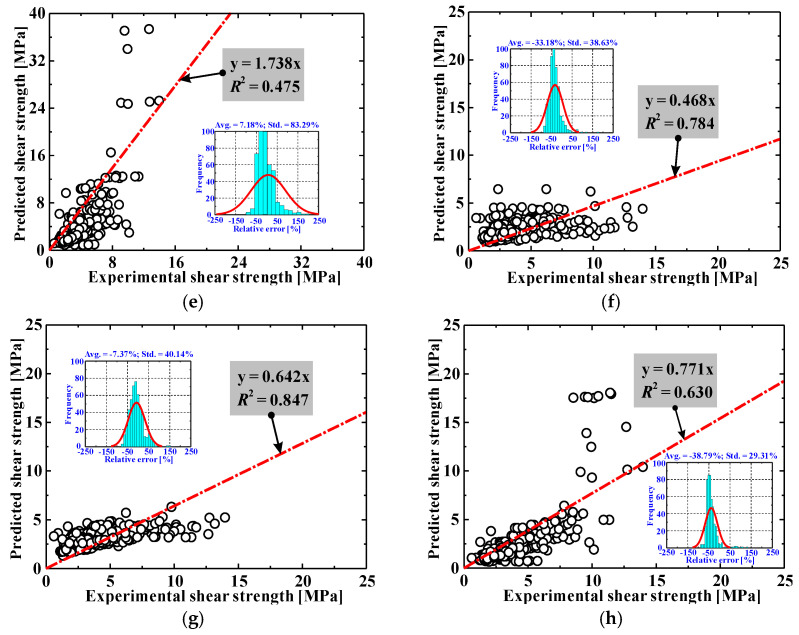
Comparisons between test results and empirical model predictions. (**a**) CECS38-2004 [41], (**b**) DAfStB-2012 [42], (**c**) *fib*-2010 [43], (**d**) Greenough and Nehdi [44], (**e**) Imam et al. [45], (**f**) Kuntia et al. [46], (**g**) Sharma [47], (**h**) Yakoub [48].

There are a considerable number of models for evaluating the shear capacity of SFRC beams. Figure 1 presents the comparison of the shear strength of the documented specimens between the measured results and the predictions using theoretical models. The models included were the codified equations in the Chinese SFRC code (CECS38-2004) [41], the German standard (DAfStB-2012) [42] and the CEB-FIP Mode Code for Structural Concrete (fib-2010) [43]. Additionally, several semi-empirical formulas suggested by Greenough and Nehdi [44], Imam et al. [45], Kuntia et al. [46], Sharma [47] and Yakoub [48] were also checked. The details of these models are summarized in Table 2. It is worth noting that most of the models were initially established based on the experimental results of normal- and high-strength SFRC beams [28], and their applicability to ultra-high-strength SFRC beams needs to be further verified. The database includes a total of 15 beams with a concrete compressive strength greater than 100 MPa. These beams were tentatively predicted by using the above empirical formulas.

From Figure 1 and Table 2, it can be concluded that the outputs of the above models vary widely. In general, the prediction accuracy of most of the models is not good enough, and some are even very poor.

The ratios of the experimental-to-predicted strength are in the range of 0.351 to 1.738, representing large scatters from one model to another. These varying degrees of incapacity can be explained by at least two factors: (i) during shear loading, the fibers’ contribution to shear is related to the effective fiber distributed area along the critical diagonal shear crack, the total amount of fibers in this area (probability-related), the fiber type and orientation, the individual fiber pull-out load–slip relationship and many other factors [9], but these factors are usually difficult to fully consider in existing design formulas, which results in great epistemic uncertainty; (ii) these models are essentially empirical, mostly calibrated with limited experimental data, which implies that the formulas are derived from a narrow range of tests and are difficult to generalize. The above results prompted this study to explore AI models.

## 3. Parameter Sensitivity Evaluation Using GRA

### 3.1. Gray Relational Analysis Principle

In the current study, a gray relational analysis (GRA) was conducted to ascertain the sensitivity/importance of different parameters for the shear capacity evaluation of SFRC beams containing no stirrups. The shear strength (*v*_u_) calculated for the database was designated as the reference matrix, *A*_0_(*j*), in which *j* = 1, 2, … *n*. The key tested parameters, including the sectional effective depth-to-width ratio (*d*/*b*), the shear span-to-effective depth ratio (*a*/*d*), the concrete’s compressive strength (*f*_c_), the maximum aggregate size (*s*_max_), the area ratio of longitudinal reinforcement (*ρ*_s_) and the fiber factor (*F*), were assigned as the comparative matrix, *A*_i_(*j*), in which, *i* = 1, 2, … *m*. The mathematical formula for constructing the relation between the reference matrix and the comparative matrix is as follows:

(1) $$\begin{array}{l} A_{0}=A_{0}(1),\;A_{0}(2),\ldots A_{0}(n)\\ A_{1}=A_{1}(1),\;A_{1}(2),\ldots A_{1}(n)\\ \ldots \\ A_{m}=A_{m}(1),\;A_{m}(2),\ldots A_{m}(n) \end{array}$$

To decrease numerical fluctuations, a normalization procedure is followed, that is, using the following operation:

(2) $$a_{i}(j)=\frac{A_{i}(j)}{\frac{1}{n}\sum_{i=1}^{n}A_{i}\left(j\right)}$$

Thus, *ξ*_i_, which represents the gray relational coefficient, is determined by:

(3) $$\xi_{i}\left[a_{0}(j),a_{i}(j)\right]=\left|\frac{\min_{i=1,n}\min_{j=1,m}\Delta_{i}(j)+\rho \max_{i=1,n}\max_{j=1,m}\Delta_{i}(j)}{\Delta_{i}+\rho \max_{i=1,n}\max_{j=1,m}\Delta_{i}(j)}\right|$$

(4) $$\Delta_{i}(j)=\left|a_{0}(j)-a_{i}(j)\right|$$

(5) $$\min_{i=1,n}\min_{j=1,m}\Delta_{i}(j)=\underset{i}{\max}\left(\underset{j}{\max}\left|a_{0}(j)-a_{i}(j)\right|\right)$$

(6) $$max_{i=1,n}\max_{j=1,m}\Delta_{i}(j)=\underset{i}{\min}\left(\underset{j}{\min}\left|a_{0}(j)-a_{i}(j)\right|\right)$$

where 0 ≤ *ρ* ≤ 1, and it frequently adopts a value of 0.50 [31].

In a GRA, the gray relational factor (*λ*) can be employed to measure the correlation degree between the reference matrix and the comparative matrix:

(7) $$\lambda =\frac{1}{n}\sum_{i=1}^{n}\xi_{i}\left[a_{0}(j),a_{i}(j)\right]\leq 1.0$$

It is noteworthy that the closer *λ* is to unity, the closer the correlations, and vice versa. In general, when *λ* is larger than 0.7, there is a strong correlation; when *λ* is smaller than 0.5, the correlation can be treated as negligible [31].

### 3.2. Assessment of Parameter Sensitivity

**Figure 2:**
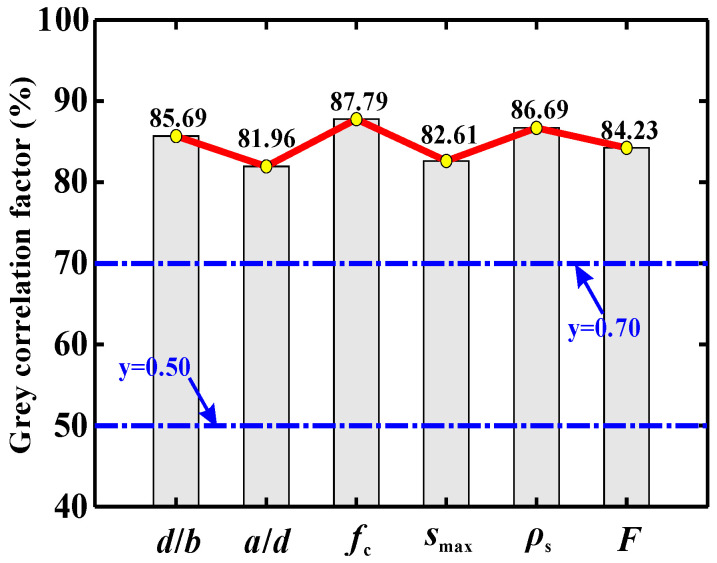
Parameter sensitivity indicated by GRA.

Figure 2 depicts the calculation results of the parameter sensitivity assessment. As indicated by the GRA, the order of importance of the parameters from large to small is: *f*_c_ > *ρ*_s_ > *d*/*b* > *F* > *s*_max_ > *a*/*d*.

The following trends can also be identified:

(a) The values of the gray relational factor (*λ*) for concrete strength *f*_c_ and maximum aggregate size *s*_max_ are 87.79% and 82.61%, respectively. The value for the fiber-related parameter *F* is 84.23%. These results suggest that, compared with steel fibers, concrete has a greater bearing on the shear strength. This is probably because concrete can resist external loads throughout the entire loading course [49]. Meanwhile, with the widening of the critical crack, the role of steel fiber will be weakened [30].
(b) The shear contribution provided by the longitudinal reinforcement cannot be ignored. This can be stressed by assigning *ρ*_s_ with secondary importance (λ = 86.69%).
(c) Additionally, the parameters *d*/*b* and *a*/*d* have notable effects on the shear capacity of SFRC beams containing no stirrups, where they are registered with *λ* values of 85.69% and 81.96%, respectively. This phenomenon is consistent with that observed in [30].

Since the *λ* values of all evaluated parameters are higher than 80%, they jointly determine the shear capacity of the SFRC beams without stirrups. Clearly, all of these parameters should be reasonably considered for a more rational and accurate shear strength prediction. These parameters are hence adopted as the inputs to the AI models, as presented in the following.

## 4. Shear Capacity Prediction Using Artificial Intelligence Models

AI models have been shown to have higher predictive accuracy than empirical models. In this study, AI models, including artificial neural network (BP-ANN), random forest (RF) and multi-gene genetic programming (MGGP), were developed to determine the shear capacity of SFRC beams without stirrups. Compared with other AI models, ANN is the most recognized at present, which reasonably considers the complex nonlinear relationship among influencing parameters. The RF model is a representative of ensemble learning AI models, which can effectively mitigate over-fitting problems. The MGGP model can provide an explicit design formula, which overcomes the black-box dilemma of AI models.

All of the modeling works were performed on the Matlab platform [50].

### 4.1. Back-Propagation Artificial Neural Network (BPANN)

**Figure 3:**
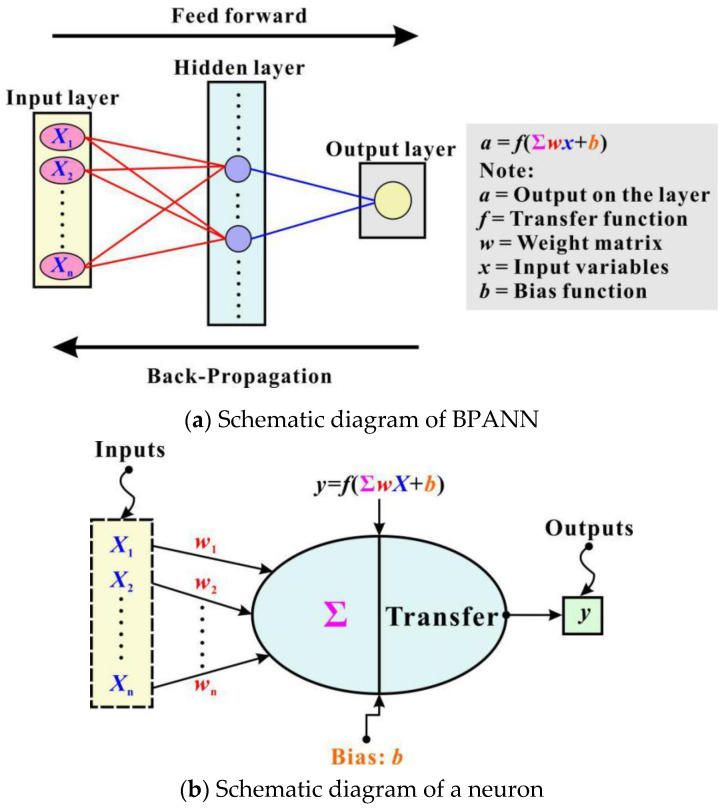
Illustration of BPANN [31].

BPANN is an AI model that tries to simulate the structural or functional aspects of biological neural networks [31]. While BPANN cannot exactly replicate the remarkable abilities of human brains, it is well suited for simulating complex processes with the proper selection of inputs and careful construction of network structures. As depicted in Figure 3a, a BPANN model has a hierarchical structure, which consists of the input layer, hidden layers and the output layer. These layers have different functions, but the basic elements of each layer, namely, neurons, are similar (see Figure 3b).

A neuron can receive information from the outside or from the neurons of the previous layer and transfer it to the next layer’s neurons. There are two main kinds of operations in neurons. The first is the weighted-sum operation, which calculates the effects of inputs together with weights on the neuron as follows:

(8) $$net_{j}=\sum_{i=1}^{n}w_{ij}x_{j}+b_{j}$$

in which *w_ij_* denotes the weight from the lower-layer neuron *i* to the upper-layer neuron *j*; *x_i_* represents neuron *i*’s output; *n* indicates the total number of *i*; and *net_j_* is the weighted sum of the upper-layer neuron *j*.

The second key operation is the sigmoid function process, which determines the signal intensity of the current neuron. The most commonly used sigmoid function has the following formula [31]:

(9) $$f\left({\mathrm{out}}_{j}\right)=\frac{1}{1+e^{-net_{j}}}$$

After completing the above two operations, the artificial neural network can provide predictive values based on the inputs. However, the outputs may not be ideal at first, and the model usually needs a correction procedure [31]. For the current BPANN, this was carried out via a back-propagation process, which is an iterative search procedure that adjusts the weights from the output layer back to the input layer until no further improvement is required. Mathematically, the correction of weights is generally in the direction of a negative error gradient [31], that is:

(10) $$\Delta w_{m}=\alpha \Delta w_{m-1}-\eta \frac{\partial E}{\partial w}$$

in which *w* is the weight between any two neurons; Δ*w_n_* and Δ*w_n_*_−1_ denote the variations in the weight *w* at *m* and *m*−1 iterations, respectively; and *η* and *α* are, respectively, the learning rate and the momentum coefficient.

In the present BPANN, a total of six input-layer neurons (variables) and one output-layer neuron were adopted. In addition, the number of neurons in the hidden layer was ascertained by seven by trial calculations, since, at this value, the model achieved an optimum result. The weights of the input layer (IWs), the biases of the input layer (IBs), the weights of the hidden layer (HWs) and the bias of the hidden layer (HB) of the BPANN model are, respectively, shown in Equations (11)–(14):

(11) $$\mathrm{IWs}=\left\{\begin{array}{cccccc} 2.8898 & 0.7396 & -0.3727 & -1.3649 & -0.5915 & 0.1703\\ -1.0552 & -1.2406 & -1.5092 & -0.9425 & 0.2803 & 1.7436\\ 0.6398 & 1.1555 & -0.3883 & -0.1229 & -0.7732 & -0.7757\\ -0.0583 & 0.9440 & 0.0584 & 2.0075 & -0.0811 & 1.3593\\ -0.3167 & -1.5704 & -0.6506 & -2.7921 & -0.1089 & -0.9115\\ 0.9003 & 4.1106 & -0.1985 & 0.0439 & -0.6175 & -0.3295\\ 0.8986 & 1.2735 & 3.2607 & -0.4838 & -1.7045 & 0.3598 \end{array}\right\}$$

(12) $${\mathrm{HBs}}^{T}=\left\{\begin{array}{ccccccc} -0.2558 & 1.1308 & 0.2721 & -0.1405 & -0.3795 & 3.5405 & 3.9120 \end{array}\right\}$$

(13) $$\mathrm{HWs}=\left\{\begin{array}{ccccccc} 0.1536 & 0.3699 & -0.5793 & -0.6016 & -0.5323 & -0.8254 & 0.2921 \end{array}\right\}$$

(14) HB={0.0033}

### 4.2. Random Forest (RF)

RF is an ensemble learning technique designed to solve regression or classification problems. This method features a special decision-making process. Typically, in RF-based regressions, the final estimate is a combination of several good predictions, rather than a single forecast.

**Figure 4:**
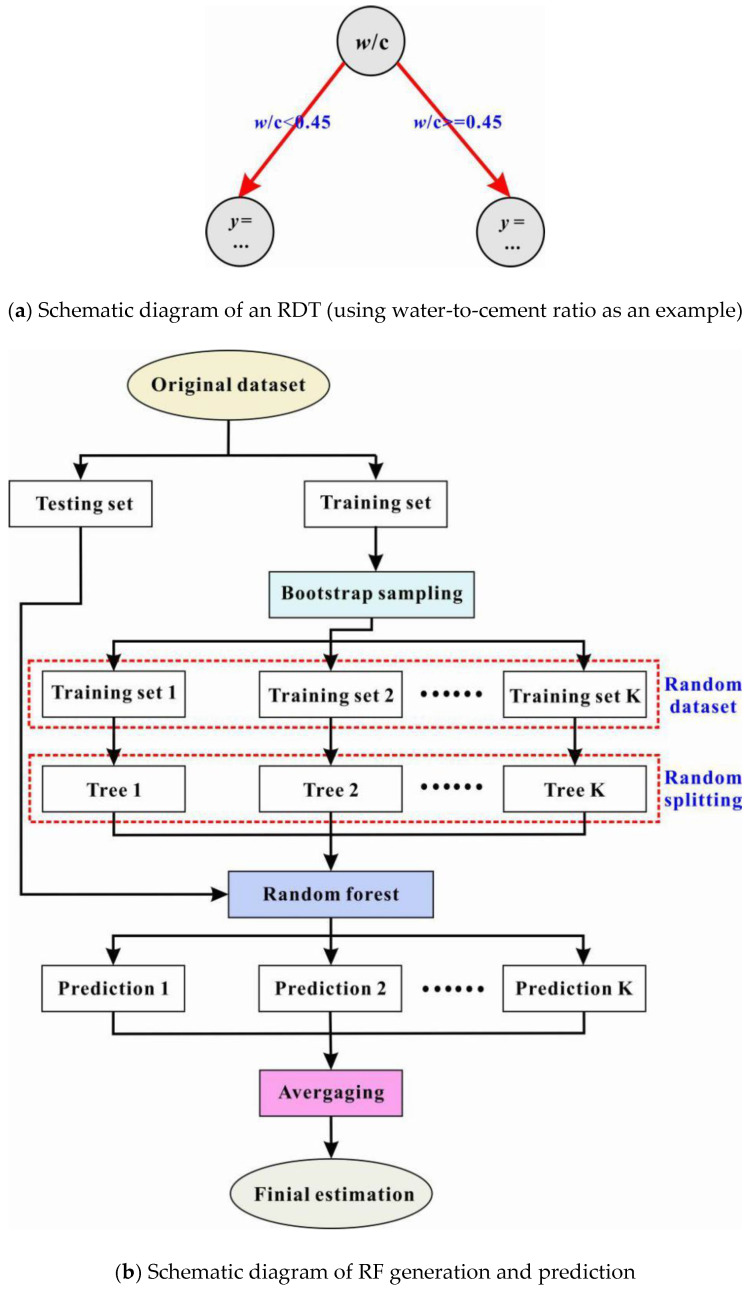
Illustration of RDT and RF [31].

Figure 4a presents an example of the typical structure of a simple regression decision tree (RDT). Specifically, the tree is utilized by reading the information of the water-to-cement ratio of a mixture to predict its compressive strength. Each circle in the graph is called a tree node, which can be understood as a character of the dataset. Typically, the topmost node in the tree is called the root node, and the bottommost nodes are defined as the terminal nodes (or leaf nodes). The arrows connecting two nodes in adjacent layers are called tree branches, and a branch represents a path choice (i.e., making a decision) for a given data point based on its feature values. For instance, the root node in Figure 4a checks the water-to-cement ratio, and if the ratio is less than 0.45, the process will move down to the left tree branch and move to the right otherwise. For a more complex RDT, operations will also start at the root node and continue along different branches. Selecting a branch can be thought of as asking a series of questions. This process of asking and answering will be repeated until the leaf node is reached. At that node, the final prediction is obtained for each data point [51].

Simply put, an RDT generates predictions by memorizing training data. This may result in an unrealistic prediction that fails to recognize the real pattern of datasets, a phenomenon known as “over-fitting” [51]. To tackle this, a combination of various decision trees can be used to make predictions. This will generate a random forest. In general, a random forest (RF) has fewer problems with over-fitting because it averages the results of a set of trees to mitigate the possible poor performance of a single tree (see Figure 4b). In addition to the split criterion and the maximum tree depth for a single RDT, an RF has several other parameters, such as the number of trees in the forest and whether bootstrapping is employed to sample trees from the forest [51].

The random forest was implemented in the Matlab toolbox M5PrimeLab [52]. The following tree configuration was used: the total number of trees in the RF was set to 100; the bootstrap sampling method was employed; each tree’s maximum depth was 9, and no pruning or smoothing was used for any tree; the minimum number of observations to be considered for splitting at a node was set to 5; the minimum number of training observations that can be represented by a leaf node was 1. Finally, the split threshold was 1 × 10^−6^.

### 4.3. Multi-Gene Genetic Programming (GP)

GP can simulate the biological evolution of a living organism. Compared with BPANN and RF, the main advantage of GP is that it can directly generate an explicit function that correlates the input variables to the output variables [53]. This capacity is mainly attributed to the tree-shaped structure of GP [53].

**Figure 5:**
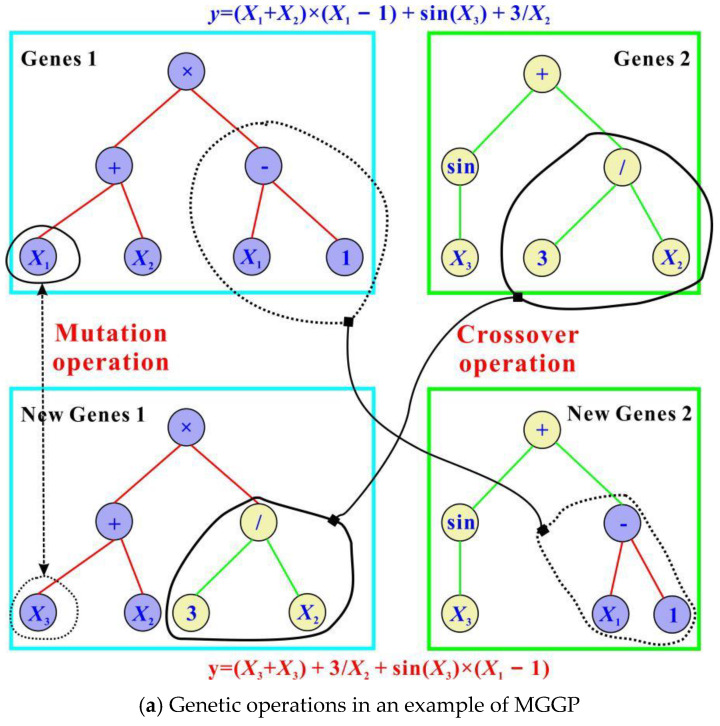

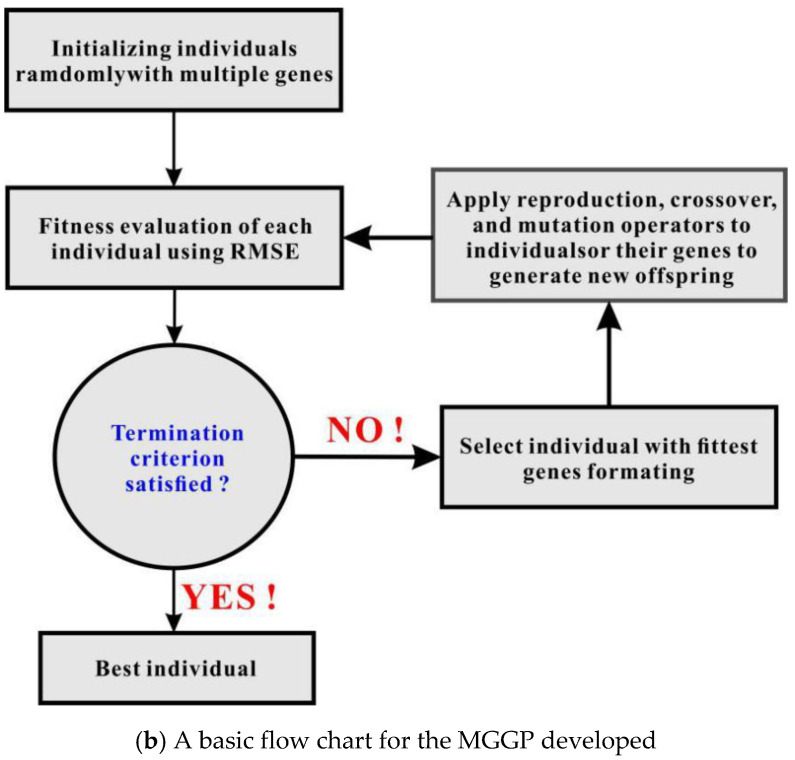
Illustration and flow chart of MGGP [31].

In traditional GP models, a single individual is commonly based on the evaluation of a tree expression. However, this cannot occur in the current multi-gene genetic programming (MGGP) due to the simultaneous existence of multiple trees. An individual evolving in an MGGP method is derived from several expression trees [50] or, in other words, from a weighted linear combination of the outputs of several traditional GP trees. To vividly illustrate MGGP’s internal structure, Figure 5a shows an example of a typical MGGP-based algorithm. Each sub-tree in this MGGP model can be treated as a “gene”. Additionally, each sub-tree consists of multiple terminals and functions. The terminals usually indicate constant coefficients and some input variables, such as *x*_1_, *x*_2_ and *x*_3_, while the functions mainly include arithmetic operations, such as addition, minus, multiplication and protected division. As can be seen from this subplot, the original expression is (*x*_1_ + *x*_2_)(*x*_1_ − 1) + sin(*x*_3_) + 3*/x*_2_. After exchanging gene segments (*x*_1_ − 1) and 3/*x*_3_ and mutating *x*_1_ as *x*_3_, the final predictive expression becomes (*x*_2_ + *x*_3_) + 3/*x*_2_+(*x*_1_ − 1)sin(*x*_3_).

Figure 5b conceptually shows the basic flow chart for the MGGP developed. The initial individuals produced by the MGGP contained randomly generated genes. These individuals were then assessed by their own fitness values, such as the root mean squared error (RMSE) applied to training and testing data [53]. By default, the smaller the RMSE, the more reliable the model. Using the fitness as a basis, individuals can be easily selected from the population through prescribed selection methods (e.g., roulette, lexictour or tournament [53]); thus, the best parents with the lowest RMSE had more opportunities to create offspring. In this process, if the termination criterion had yet to be satisfied, genetic operators such as crossover and mutation were performed again to generate new individuals. Their performance was then evaluated with regard to prediction errors, and a new generation was created by replacing some or all of the individuals in the original population with the new individuals. This process was repeated until the termination criterion was met.

The current MGGP model was developed using MATLAB [50] in conjunction with the GPTIPS toolbox [53]. The optimal parameters used in the MGGP model are listed in Table 3. As presented, quite a few parameters were involved in the analysis. In this study, the values of some parameters were referred to [54], including the function set, elitism and the probability of crossover, mutation and reproduction events. Other parameters, however, were determined through extensive trial-and-error, which were mainly related to the size of the population, the generation number, the maximum depth of the tree and the maximum allowable number of genes. Using the population size and the generation number as an example, these parameters were set as the optimal trade-off between solution complexity and running time.

Table 4 shows the MGGP model’s individual genes. These genes were summed automatically and simplified by the toolbox of the GITIPS, which generated the following equation:

(15) y=Bias+Gene 1+Gene 2+ … +Gene 10

### 4.4. Prediction Results and Discussion

The database about the shear strength was randomly divided into a training set and a testing set in a ratio of 3:1. The data in the training set and the testing set were used for AI model learning and prediction, respectively.

Aiming to evaluate the predictive responses of the BPANN, RF and MGGP models implemented in the present study, the correlation coefficient (*R*^2^), the root mean squared error (RMSE) and the mean absolute percentage error (MAPE) were used to reflect the prediction errors. A lower RMSE or MAPE value indicates a better prediction accuracy, and a higher *R*^2^ value corresponds to a closer fit [31]. These metrics can be calculated using the following equations:

(16) $$\mathrm{RMSE}=\sqrt{\frac{\sum_{i}^{N}{\left(p_{i}-x_{i}\right)}^{2}}{N}}$$

(17) $$\mathrm{MAPE}=\frac{1}{N}\sum_{i}^{N}{\left(\frac{p_{i}-x_{i}}{x_{i}}\right)}^{2}$$

(18) $$R^{2}=1-\frac{\sum_{i}^{N}{\left(p_{i}-x_{i}\right)}^{2}}{\sum_{i}^{N}{\left(x_{i}-x^{\mathrm{avg}}\right)}^{2}}$$

where *p* and *x* are the predicted output matrix and the measured output matrix, respectively; *x*_avg_ is the average value of the measured outputs; and *N* is the total number of data in the training or testing sets.

**Figure 6:**
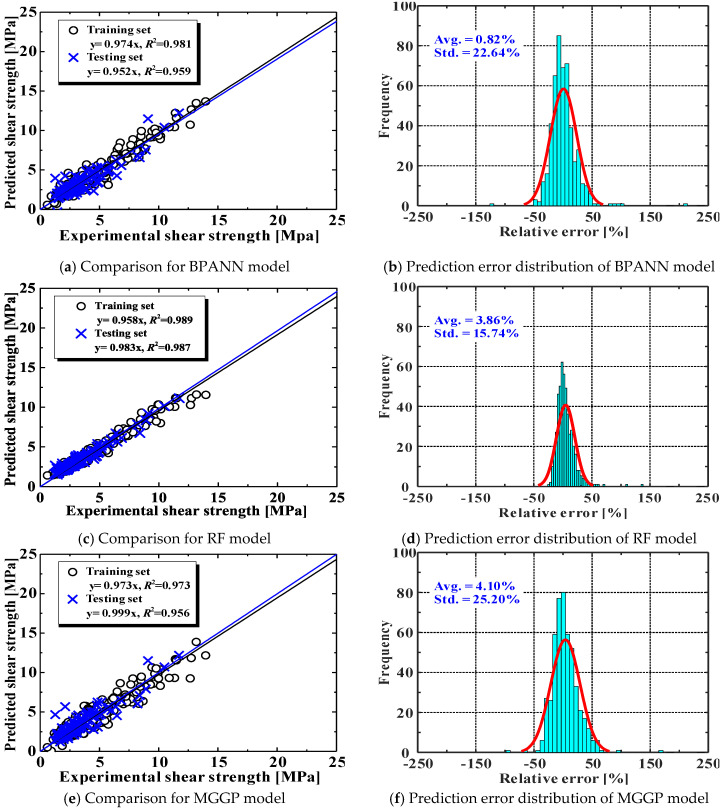
Comparisons between experimental results and predictions using AI models.

Comparisons between the experimental data and the predictions of the AI models are shown in Figure 6. Evidently, all three AI models performed well in both training and testing datasets, and their *R*^2^ values were all higher than 0.95. The RF model showed slightly higher accuracy than the other models. It can also be observed from Figure 6b that the error frequency of the RF model conformed to a normal distribution.

**Figure 7:**
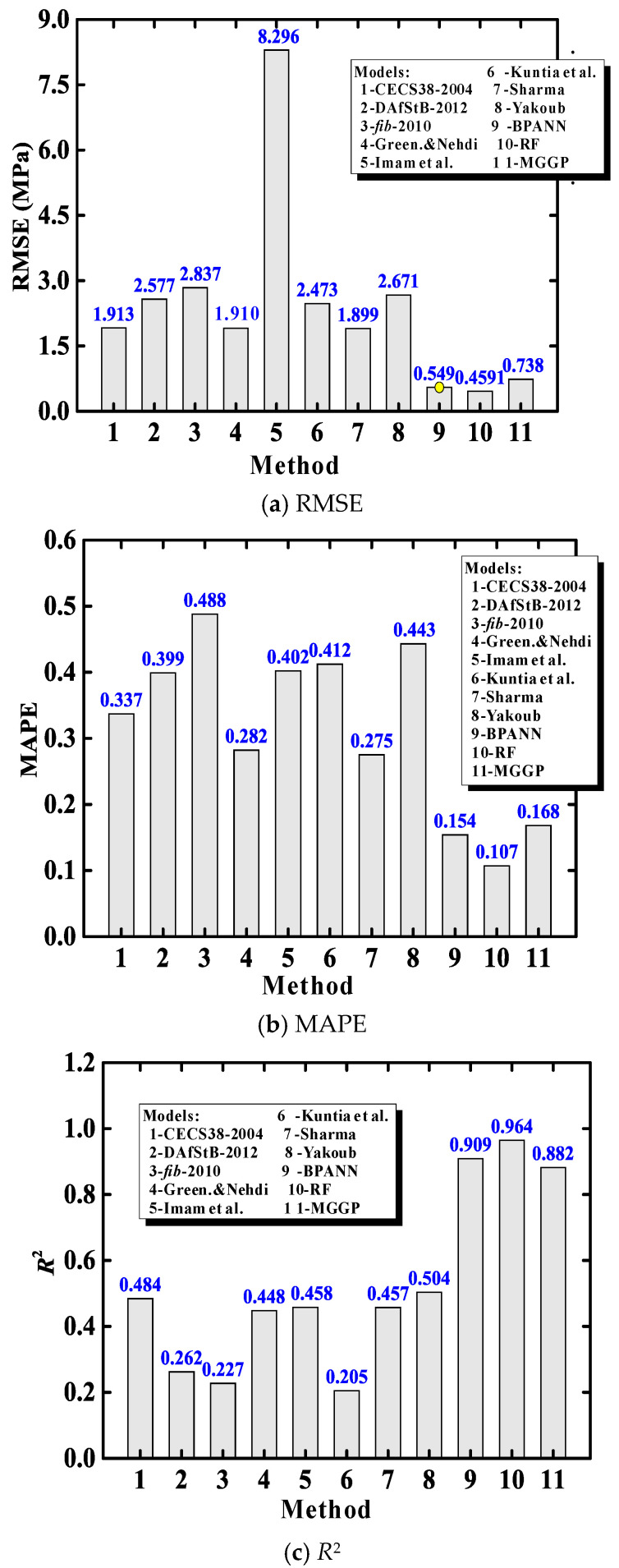
Comparison of prediction performance of different models.

Figure 7 compares the predictive performance between AI models (Methods 9–11) and the previously evaluated empirical equations (Methods 1–8). The results clearly indicate that the AI models had much higher accuracy than the empirical models. It is further confirmed that the RF model slightly outperformed the other models, because it has smaller RMSE and MAPE values. This advantage comes from the strength of ensemble learning approaches [33], which are often superior to standalone AI models (such as BPANN). Nevertheless, the MGGP model with good accuracy provides closed-form expressions.

## 5. Parametric Study

After the AI models were established, the effects of different key parameters on the SFRC beam shear strength could be further studied. By verifying whether the AI models could duplicate the experimentally observed influences of the parameters, the interpretability of the AI models could be effectively examined. The parametric study was carried out by varying one parameter’s value while fixing the others.

Table 5 lists the numerical variation trend for each influential factor. Note that the reference sample shown in this table is an SFRC beam with [*d*/*b*, *a*/*d*, *f*_c_, *s*_max_, *ρ*_s_, *F*] = [2, 3.0, 50, 10, 2.5%, 50%]. Compared with the statistical indicators summarized in Table 1, it is not difficult to find that the parameter values adopted by this beam are quite close to the average values in the database. In addition, the maximum and minimum parameter values for each beam in Table 5 are also close to the upper and lower limits in the test database. Thus, we believe that the selected beams in Table 5 still fall within the applicability of the database.

### 5.1. Influence of the SFRC’s Compressive Strength

Previous studies [30] have shown that the shear strength of SFRC beams without stirrups is greatly affected by the concrete’s compressive strength: i.e., the shear strength *v*_u_ increases with an increase in the concrete strength *f*_c_. It is evident from the predictions in Table 5, Group I, that all eight empirical formulas (Methods 1–8), together with the developed AI models (Methods 9–11), correctly predict this trend.

**Figure 8:**
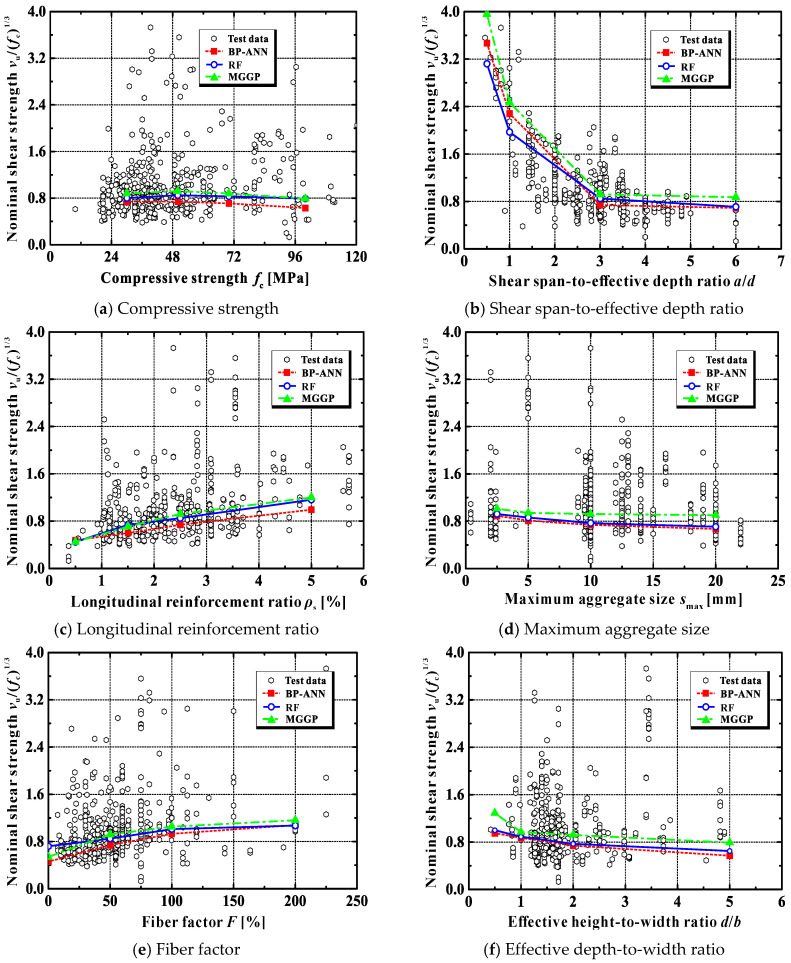
Main parameters affecting the shear strength of SFRC beams without stirrups.

Figure 8a further displays the experimental and predicted relationships between the nominal shear strength *v*_u_/(*f*_c_)^1/3^ and the compressive strength *f*_c_. As can be seen from this plot, once it is normalized with respect to the cubic root of *f*_c_ (i.e., (*f*_c_)^1/3^), the shear strength of the tested beams is no longer sensitive to the concrete strength. This implies that the prediction model to predict the shear strength of SFRC beams without stirrups should be based on the cubic root of the compressive strength of SFRC, which is consistent with the recommendation in [31]. Clearly, the predictions obtained using the AI models can well reflect this trend.

### 5.2. Influence of the Shear Span-to-Effective Depth Ratio

The test data presented in Figure 8b show the influence of the shear span-to-effective depth ratio *a*/*d* on the nominal shear strength of SFRC beams without stirrups. As the value of *a*/*d* decreases, the shear strength increases notably, especially when *a*/*d* is less than about 3.0. This result is in agreement with the observations in [31].

In addition, it can be seen from Group II in Table 5 that code-based expressions (CECS38-2004 [41], DAfStB-2012 [42] and fib-2010 [43]) cannot capture the influence of *a*/*d*. The models proposed by Imam et al. [45] and Yakoub [48] can reproduce the decreasing trend of the shear capacity against *a*/*d*, but these models overestimate the nominal shear strength of deep SFRC beams with *a*/*d* less than 1.0. The shear strengths predicted by the BPANN, RF and MGGP models agree well with the experimental results and reflect the influence of *a*/*d* in a wide range, as shown in Figure 8b.

### 5.3. Effect of the Area Ratio of Longitudinal Reinforcement

For ordinary concrete beams, increasing the longitudinal reinforcement ratio will increase their shear capacities [55], because: (a) the dowel action can be significantly improved, (b) the contribution of uncracked concrete in the compression zone also increases as a result of the equilibrium requirement in the cross-section, and (c) the arching action is similarly more mobilized. The test data shown in Figure 8c suggest that *ρ*_s_ has a positive influence on the beams’ shear capacity.

The predicted shear strengths for beams with a longitudinal reinforcement ratio between 0.5% and 5.0% are shown in Group III in Table 5. As can be seen, the AI models corroborate the effect of beam tensile reinforcement, while some formulas (CECS38-2004 [41], Kuntia et al. [46] and Sharma [47]) completely ignore its significant role.

### 5.4. Effect of the Maximum Aggregate Size

The use of larger coarse aggregates in ordinary concrete often improves the aggregate interlocking capacity and, further, the shear capacity of concrete beams [56]. However, this mechanism might not be true for SFRC beams. As can be seen from the test data in Figure 8d, the nominal shear strength presents a decreasing trend as the maximum aggregate size increases. This phenomenon may be because the use of larger aggregates usually results in a change in fiber orientation, which greatly affects the tensile capacity of SFRC.

None of the empirical models can predict the above-mentioned effect of coarse aggregate size (see Group IV of Table 5). Note that only the formulas proposed by Imam et al. [45] and Yakoub [48] consider the influence of *s*_max_ on the shear capacity of SFRC beams without stirrups. However, their predicted results show an increasing trend as the aggregate size increases, which is opposite to experimental observations.

In contrast, the BPANN, RF and MGGP models are able to represent the influence of *s*_max_ on the shear strength, as presented in Figure 8d.

### 5.5. Effect of the Fiber Factor

As mentioned previously, the addition of steel fibers is efficient in boosting the structural performance of ordinary concrete members. The experimental results in Figure 8e confirm this merit. As the fiber factor *F* increases, the beam shear strength rises, too. Furthermore, as can be seen in Group V, Table 5, except for the model proposed by Sharma [47], both the empirical and AI models can faithfully reproduce the influence of steel fibers.

It is also evident from Figure 8e that the shear strength of SFRC beams without stirrups increases significantly with the increase in the fiber factor *F* when *F* is below a threshold of about 50%. Beyond that, this positive effect gradually diminishes. When *F* exceeds 100%, the effect disappears. This suggests that the content of steel fiber in SFRC beams should be controlled to avoid wasting materials.

### 5.6. Effect of the Sectional Effective Depth-to-Width Ratio

Figure 8f displays the impact of the effective depth-to-width ratio *d*/*b* on the nominal shear strength of SFRC beams without stirrups. As *d*/*b* increases, a reduction in the beam shear capacity is apparent. There are two main reasons for this. The first is due to the size effect. That is, if the beam width *b* remains unchanged, an increase in *d*/*b* means an increase in the effective depth *d*. In this case, the concrete member’s strength will scale down according to Bazant’s size-effect law [57]. The second reason is the change in the stress condition of concrete. An increase in *d*/*b* corresponds to a decrease in *b* when *d* remains constant. Thus, the stress condition of concrete progressively evolves from the plane strain state to the plane stress state, which reduces the concrete’s capacity to resist external loads, as explained in [58].

From the simulation results of Group VI listed in Table 5, with the exception of the empirical models CECS38-2004 [41], Kuntia et al. [46] and Sharma [47], all other models can realistically represent the influence of the *d*/*b* ratio.

## 6. Conclusions

Existing empirical models for predicting the shear capacity of SFRC beams containing no stirrups are not very accurate. In this study, three typical AI models were leveraged to improve the prediction accuracy and to explain the experimentally obtained test results. The following important findings can be obtained from this study:

(1) The empirical strength models evaluated in this paper cannot predict with desirable accuracy the shear bearing capacity of SFRC beams without stirrups. There are a number of reasons for this, including the inadequate account of the role of steel fibers (such as the effective fiber distributed area along the critical diagonal shear crack, the total amount of fibers in this area, the fiber type and orientation, and the individual fiber pull-out load–slip relationship) and the limited database that the models were derived from.
(2) The GRA results indicate that the shear strength of the beams depends crucially on the following parameters: the material properties of concrete, the amount of longitudinal reinforcement, the attributes of steel fibers, and the geometrical and loading characteristics of SFRC beams in shear. The *λ* values of these parameters are all greater than 80%, indicating that all of these parameters should be considered for a more rational prediction of beam shear strength. Unfortunately, none of the empirical models evaluated take these parameters into full account.
(3) The three AI models—BPANN, RF and MGGP—are effective in predicting the shear capacity of SFRC beams without stirrups. Their predictive performance is excellent, with all *R*^2^ values higher than 0.95. By contrast, RF slightly surpasses BPANN and MGGP (mainly because RF is an ensemble learning method, which combines the results of multiple weak learners), while MGGP provides an unambiguous design expression. The AI models fit the experimental data in both the training and testing sets, showing good generalization capacity within the range of the data collected.
(4) The AI models were used to perform a parametric study to strengthen support for experimental trends. The results show that these models reveal the potential effects of all of the important factors affecting the shear capacity, and these effects can be reasonably explained. Therefore, the developed AI models can be used as fast, accurate and simulation-free tools for designing SFRC beams without stirrups.

An application issue of AI models is finally discussed here. AI-based models, however accurate, are not intended to discredit conventional mechanics-based methods. In fact, some have expressed legitimate concern that the potential benefits of AI models to structural engineering are being overhyped. It is therefore critical that developers of AI models communicate openly and honestly with users about limitations and potential pitfalls. The current authors believe that AI models are just alternative tools that are expected to support/complement conventional methods. In the long term, the inclusion of basic data science courses in civil engineering education could increase the number of practitioners that have a working knowledge of AI algorithms. Nevertheless, there are some promising areas (such as the problem at hand) where AI models can provide meaningful benefits. It should be borne in mind that effective implementation requires more genuinely representative datasets and a reasonable trade-off between the benefits and drawbacks of using AI models.

## Figures and Tables

**Table 1:** Descriptive statistics of variables in the database.

Parameter	*d/b*	*a*/*d*	*f*_c_ (MPa)	*s*_max_ (mm)	*ρ*_s_ (%)	*F* (%)	*v*_u_ (MPa)
Maximum	4.90	6.00	215.0	22.0	5.72	285.75	13.96
Mean	1.81	2.92	49.0	10.7	2.46	53.95	3.64
Minimum	0.42	0.46	9.8	0.4	0.37	7.50	0.60
Standard deviation	0.77	0.98	25.2	5.1	1.01	35.96	2.14
Standard error	0.03	0.04	1.1	0.2	0.05	1.63	0.10
Median	1.57	3.00	40.3	10.0	2.54	48.75	3.01
Mode	1.26	2.00	33.2	10.0	3.09	60.00	2.61
Kurtosis	3.99	0.37	7.8	−0.1	0.98	7.14	4.72
Skewness	1.94	0.00	2.2	0.0	0.77	2.02	2.06

**Table 2:** Empirical shear strength models for SFRC beams without stirrups.

Reference	Equation
CECS38-2004 [41]	$v_{u}=\frac{1.75}{1+a/d}f_{t}(1+\beta_{v}V_{f}\frac{l_{f}}{d_{f}})$ $\frac{a}{d}=\min[\max(\frac{a}{d},\;1.5),\;3.0]$ $\beta_{v}=\{\begin{array}{c} 0.70\;\;\;\;\;\;\;\;\;\;\;\;\;\;\;\;\;\;\mathrm{shear}-\mathrm{flat}\;\mathrm{fibers}\\ 0.50\;\;\;\;\mathrm{shear}-\mathrm{cut}\;\mathrm{profied}\;\mathrm{fibers}\\ 0.60\;\;\;\;\;\;\;\;\mathrm{cut}-\mathrm{off}\;\mathrm{profied}\;\mathrm{fibers}\\ 0.90\;\;\;\;\;\;\mathrm{mill}-\mathrm{cut}\;\mathrm{profied}\;\mathrm{fibers} \end{array}$ $f_{t}=\{\begin{array}{c} 0.3{\left(f_{c}-8\right)}^{\frac{2}{3}}\;\;\;\;\;\;\;\;\;\;\;\;\;\;\;\;\;\;\;\;\;\;\;\;\;\;\;\;\;\;\;\;\;\;\;\;\;\;\;\;\;\;\;\;\;\;\;\;\;\;\;\;\;\;\;\;\;\;\;\;\;\;\;\;\;\;f_{c}\leq 58\;\mathrm{MPa}\;\\ 2.12\;\ln(1+0.1f_{c})\;\;\;\;\;\;\;\;\;\;\;\;\;\;\;\;\;\;\;\;\;\;\;\;\;\;\;\;\;\;\;\;\;\;\;\;\;\;\;\;\;\;\;\;\;\;\;\;\;\;\;\;\;\;\;\;f_{c}>58\;\mathrm{MPa}\; \end{array}$
DAfStB-2012 [42]	$v_{u}=v_{c}+v_{f}$ $v_{c}=0.12k{\left[\rho_{s}(f_{c}-8)\right]}^{\frac{1}{3}}$ $k=1+\sqrt{\frac{200}{d}}$ (d: in mm) $v_{f}=0.68f_{\mathrm{ctR},u}^{f}\frac{h}{d}$ $f_{\mathrm{ctR},u}^{f}=0.185k_{G}^{f}f_{\mathrm{cfIk},\;l2}^{f}$ $k_{G}^{f}=\min(1.0+0.5A_{\mathrm{ct}}^{f},\;1.7)$ $A_{\mathrm{ct}}^{f}=b/1000\times \min(d,\;1500)/1000$ (b: in mm) $f_{\mathrm{cfIk},\;l2}^{f}=0.63\sqrt{f_{c}/0.85}+2.88\times 10^{-3}F\sqrt{f_{c}/0.85}+5.20\times 10^{-4}F$
*Fib*-2010 [43]	$v_{u}=0.12k{\left[\rho_{s}(1+7.5\frac{f_{\mathrm{ctR},u}^{f}}{f_{t}})(f_{c}-8)\right]}^{\frac{1}{3}}$ $f_{t}=\{\begin{array}{c} 0.3{\left(f_{c}-8\right)}^{\frac{2}{3}}\;\;\;\;\;\;\;\;\;\;\;\;\;\;\;\;\;\;\;\;\;\;\;\;\;\;\;\;\;\;\;\;\;\;\;\;\;\;\;\;\;\;\;\;\;\;\;\;\;\;\;\;\;\;\;\;\;\;\;\;\;\;\;\;\;\;\;f_{c}\leq 58\;\mathrm{MPa}\;\\ 2.12\;\ln(1+0.1f_{c})\;\;\;\;\;\;\;\;\;\;\;\;\;\;\;\;\;\;\;\;\;\;\;\;\;\;\;\;\;\;\;\;\;\;\;\;\;\;\;\;\;\;\;\;\;\;\;\;\;\;\;\;\;\;\;\;\;\;f_{c}>58\;\mathrm{MPa}\; \end{array}$
Greenough and Nehdi [44]	$v_{u}=0.35(1+\sqrt{\frac{400}{d}}){\left(f_{c}\right)}^{0.18}{\left[\rho_{s}\frac{d}{a}(1+0.01F)\right]}^{0.4}+0.01531F$ (d: in mm)
Imam et al. [45]	$v_{u}=\frac{0.70}{\sqrt{1+\frac{d}{25s_{\max}}}}{\left(0.01\rho_{s}\right)}^{1/3}\{f_{c}{}^{0.44}[1+{\left(0.01F\right)}^{1/3}]+870\sqrt{\frac{0.01\rho_{s}}{{\left(\frac{a}{d}\right)}^{5}}}\}$ (d: in mm)
Kuntia et al. [46]	$v_{u}=(0.167+0.25F/100)\sqrt{f_{c}}$
Sharma [47]	$v_{u}=0.533{\left(\frac{d}{a}\right)}^{\frac{1}{4}}\sqrt{f_{c}}$
Yakoub [48]	$v_{u}=\{\begin{array}{c} 0.83\xi {\left(0.01\rho_{s}\right)}^{1/3}(\sqrt{f_{c}}+249.28\sqrt{\frac{0.01\rho_{s}}{{\left(\frac{a}{d}\right)}^{5}}}+0.162R_{f}V_{f}\sqrt{f_{c}}\frac{l_{f}}{d_{f}})\;\;\;\;\;\;\;\;a/d>2.5\\ 0.83\xi {\left(0.01\rho_{s}\right)}^{1/3}(\sqrt{f_{c}}+249.28\sqrt{\frac{0.01\rho_{s}}{{\left(\frac{a}{d}\right)}^{5}}}+0.405R_{f}V_{f}\sqrt{f_{c}}\frac{l_{f}}{d_{f}}\frac{d}{a})\;\;\;\;\;\;a/d\leq 2.5 \end{array}$ $\xi =\frac{1}{\sqrt{1+\frac{d}{25s_{\max}}}}$ (d: in mm) $R_{f}=\{\begin{array}{c} 0.83\;\;\;\;\;\;\;\;\;\mathrm{crimped}\;\mathrm{fibers}\\ 1.00\;\;\;\;\;\;\;\;\;\mathrm{hooked}\;\mathrm{fibers}\\ 0.91\;\;\;\;\;\;\;\;\;\mathrm{rounded}\;\mathrm{fibers} \end{array}$

Note: both *ρ*_s_ and *F* in above equations are expressed as %.

**Table 3:** Parameter settings for the MGGP model.

Parameter Definition	Setting
Population size	1000
Number of generations	1000
Max number of genes	10
Max genes’ tree depth	6
Function set	plus, minus, times, divide, sqrt, square, cube, sin
Tournament size	20
Elitism	5% of population
Probability of crossover event	0.85
Probability of mutation event	0.10
Probability of reproduction event	0.05

**Table 4:** Individual genes in the best MGGP model.

Term	Value
Bias	53.4
Gene 1	$-1.141\;{\left[x_{6}+x_{2}\sqrt{x_{1}}\right]}^{1/4}$
Gene 2	$-35.32x_{1}^{1/4}$
Gene 3	$-(x_{1}+2\sqrt{x_{1}})\sqrt{\sin(x_{1}^{2})+x_{2}^{2}+\sqrt{x_{2}}}+0.003772(3x_{1}+x_{3}+x_{1}x_{2})$
Gene 4	$-0.008006x_{4}{}^{1/4}(2x_{2}+x_{6}-\sin(x_{1}x_{4})+2x_{1}x_{2})$
Gene 5	$6.794\sqrt{4x_{1}x_{2}+x_{2}\sqrt{x_{1}}}$
Gene 6	$28.78{\left(x_{2}^{3}+x_{2}\right)}^{1/4}$
Gene 7	$-2.88x_{1}^{1/4}x_{2}x_{5}^{1/8}$
Gene 8	$0.6659\sqrt{x_{5}(x_{3}+\sin x_{4})-x_{5}^{3/2}}$
Gene 9	$-59.93\sqrt{x_{2}}$
Gene 10	$0.6062\sqrt{x_{6}+\sqrt{x_{2}}}$

Note: *x*_1_, *x*_2_, *x*_3_, *x*_4_, *x*_5_ and *x*_6_ are, respectively, *d*/*b*, *a*/*d*, *f*_c_, *s*_max_, *ρ*_s_ and *F.*

**Table 5:** Shear strengths of SFRC beams without stirrups predicted by different models.

Group	Influence Parameters	*v*_u_ Predicted by Different Methods										
No.	[*d*/*b*, *a*/*d*, *f*_c_, *s*_max_, *ρ*_s_, *F*]	1	2	3	4	5	6	7	8	9	10	11
I	[2, 3.0, 30, 10, 2.5%, 50%]	1.55	1.44	1.22	2.18	2.14	1.60	2.22	1.27	2.29	2.49	2.74
	[2, 3.0, 50, 10, 2.5%, 50%]	2.38	1.81	1.45	2.31	2.39	2.06	2.86	1.53	2.74	3.15	3.37
	[2, 3.0, 70, 10, 2.5%, 50%]	2.89	2.10	1.64	2.41	2.60	2.44	3.39	1.74	2.92	3.43	3.64
	[2, 3.0, 100, 10, 2.5%, 50%]	3.34	2.45	1.89	2.52	2.85	2.92	4.05	2.01	2.93	3.67	3.68
II	[2, 0.5, 50, 10, 2.5%, 50%]	3.81	1.81	1.45	3.93	100.1	2.06	4.48	35.05	12.80	11.49	14.59
	[2, 1.0, 50, 10, 2.5%, 50%]	3.81	1.81	1.45	3.17	18.73	2.06	3.77	7.21	8.40	7.26	9.11
	[2, 3.0, 50, 10, 2.5%, 50%]	2.38	1.81	1.45	2.31	2.39	2.06	2.86	1.53	2.74	3.15	3.37
	[2, 6.0, 50, 10, 2.5%, 50%]	2.38	1.81	1.45	1.94	1.47	2.06	2.41	1.22	2.55	2.62	3.22
III	[2, 3.0, 50, 10, 0.5%, 50%]	2.38	1.41	0.85	1.58	1.04	2.06	2.86	0.77	1.77	1.65	1.67
	[2, 3.0, 50, 10, 1.5%, 50%]	2.38	1.66	1.22	2.03	1.81	2.06	2.86	1.22	2.20	2.72	2.61
	[2, 3.0, 50, 10, 2.5%, 50%]	2.38	1.81	1.45	2.31	2.39	2.06	2.86	1.53	2.74	3.15	3.37
	[2, 3.0, 50, 10, 5.0%, 50%]	2.38	2.07	1.83	2.81	3.60	2.06	2.86	2.13	3.66	4.29	4.44
IV	[2, 3.0, 50, 2.5, 2.5%, 50%]	2.38	1.81	1.45	2.31	1.42	2.06	2.86	0.91	3.26	3.38	3.72
	[2, 3.0, 50, 5.0, 2.5%, 50%]	2.38	1.81	1.45	2.31	1.88	2.06	2.86	1.20	3.00	3.18	3.47
	[2, 3.0, 50, 10, 2.5%, 50%]	2.38	1.81	1.45	2.31	2.39	2.06	2.86	1.53	2.74	2.85	3.37
	[2, 3.0, 50, 20, 2.5%, 50%]	2.38	1.81	1.45	2.31	2.88	2.06	2.86	1.84	2.47	2.63	3.30
V	[2, 3.0, 50, 10, 2.5%, 0%]	1.59	1.65	1.38	1.32	1.83	1.18	2.86	1.44	1.69	2.66	1.98
	[2, 3.0, 50, 10, 2.5%, 50%]	2.38	1.81	1.45	2.31	2.39	2.06	2.86	1.53	2.74	3.15	3.37
	[2, 3.0, 50, 10, 2.5%, 100%]	3.17	1.98	1.51	3.27	2.54	2.95	2.86	1.62	3.41	3.71	3.86
	[2, 3.0, 50, 10, 2.5%, 200%]	4.76	2.30	1.62	5.10	2.72	4.72	2.86	1.79	3.98	3.94	4.27
VI	[0.5, 3.0, 50, 10, 2.5%, 50%]	2.38	2.39	2.04	3.09	3.26	2.06	2.86	2.09	3.51	3.69	4.74
	[1.0, 3.0, 50, 10, 2.5%, 50%]	2.38	2.03	1.69	2.63	2.88	2.06	2.86	1.84	3.21	3.29	3.59
	[2.0, 3.0, 50, 10, 2.5%, 50%]	2.38	1.81	1.45	2.31	2.39	2.06	2.86	1.53	2.74	2.85	3.37
	[5.0, 3.0, 50, 10, 2.5%, 50%]	2.38	1.67	1.25	2.03	1.73	2.06	2.86	1.10	2.08	2.40	2.93

Note: (1) In the calculations, each beam’s sectional width and concrete cover are, respectively, assumed to be 200 mm and 25 mm; (2) Models 1–8 are those proposed by CECS38-2004 [41], DAfStB-2012 [42], *fib*-2010 [43], Greenough and Nehdi [44], Imam et al. [45], Kuntia et al. [46], Sharma [47] and Yakoub [48], respectively. Models 9–11 are the established BPANN, RF and MGGP models.

## Data Availability

The data presented in this study are available upon request from the corresponding author.

## Supplementary Materials

The following supporting information can be downloaded at: https://www.mdpi.com/1996-1944/15/7/2407/s1, Table S1: Appendix File Database information of experimental SFRC beams without stirrups [28].
