# Supplementary material for: Evaluation of Shear Capacity of Steel Fiber Reinforced Concrete Beams without Stirrups Using Artificial Intelligence Models
Source: Materials (Basel). 2022 Mar 24;15(7):2407. doi: 10.3390/ma15072407 (PMC9254746; doi:10.3390/ma15072407)
Supplement: Supplementary file 1 [file materials-15-02407-s001.zip › Supplementary data.pdf]

Table S1. Appendix File Database information of experimental SFRC beams without stirrups
(This database was originally collected by Lantsoght [28], it is rephrased the data structure in this work)

Source	b [mm]	h [mm]	d [mm]	a/d	f_c [MPa]	s_{max} [mm]	ρ_s [%]	Fiber type	ρ_f	f_f [MPa]	V_f [%]	d_f [mm]	L_f/d_f	F [%]	V_u [kN]
	150	300	251	3.49	28.1	12.5	2.67	Hooked	1.00	1100	0.75	0.55	65	48.75	113.8
	150	300	251	3.49	25.3	12.5	2.67	Hooked	1.00	1100	0.75	0.55	65	48.75	79.9
	150	300	251	3.49	27.9	12.5	2.67	Hooked	1.00	1100	1.00	0.55	65	65.00	110.0
	150	300	251	3.49	26.2	12.5	2.67	Hooked	1.00	1100	1.00	0.55	65	65.00	124.3
	150	300	251	3.49	28.1	12.5	2.67	Hooked	1.00	1100	1.50	0.55	65	97.50	111.9
	150	300	251	3.49	27.3	12.5	2.67	Hooked	1.00	1100	1.50	0.55	65	97.50	131.8
	150	300	251	3.49	27.5	12.5	2.67	Hooked	1.00	1050	0.50	0.75	80	40.00	65.6
	150	300	251	3.49	24.9	12.5	2.67	Hooked	1.00	1050	0.50	0.75	80	40.00	78.0
	150	300	251	3.49	27.8	12.5	2.67	Hooked	1.00	1050	0.75	0.75	80	60.00	91.9
	150	300	251	3.49	27.3	12.5	2.67	Hooked	1.00	1050	0.75	0.75	80	60.00	102.5
	150	300	251	3.49	26.3	12.5	2.67	Hooked	1.00	1050	1.00	0.75	80	80.00	116.8
	150	300	251	3.49	27.1	12.5	2.67	Hooked	1.00	1050	1.00	0.75	80	80.00	105.5
	150	300	251	3.49	53.4	12.5	2.67	Hooked	1.00	1100	0.75	0.55	65	48.75	113.8
	150	300	251	3.49	54.1	12.5	2.67	Hooked	1.00	1100	0.75	0.55	65	48.75	126.6
Singh & Jain	150	300	251	3.49	53.2	12.5	2.67	Hooked	1.00	1100	1.00	0.55	65	65.00	145.0
	150	300	251	3.49	55.3	12.5	2.67	Hooked	1.00	1100	1.00	0.55	65	65.00	166.5
	150	300	251	3.49	64.6	12.5	2.67	Hooked	1.00	1100	1.50	0.55	65	97.50	196.2
	150	300	251	3.49	59.9	12.5	2.67	Hooked	1.00	1100	1.50	0.55	65	97.50	160.8
	150	300	251	3.49	47.8	12.5	2.67	Hooked	1.00	1050	0.50	0.75	80	40.00	128.5
	150	300	251	3.49	49.5	12.5	2.67	Hooked	1.00	1050	0.50	0.75	80	40.00	152.9
	150	300	251	3.49	55.3	12.5	2.67	Hooked	1.00	1050	0.75	0.75	80	60.00	147.3
	150	300	251	3.49	56.4	12.5	2.67	Hooked	1.00	1050	0.75	0.75	80	60.00	178.9
	150	300	251	3.49	53.4	12.5	2.67	Hooked	1.00	1050	1.00	0.75	80	80.00	128.8
	150	300	251	3.49	51.0	12.5	2.67	Hooked	1.00	1050	1.00	0.75	80	80.00	157.8
	150	300	251	3.49	27.8	12.5	2.67	Crimped	0.75	1025	1.00	0.60	50	37.50	79.5
	150	300	251	3.49	27.2	12.5	2.67	Crimped	0.75	1025	1.00	0.60	50	37.50	78.8
	150	300	251	3.49	27.6	12.5	2.67	Crimped	0.75	1050	1.00	0.70	85	63.75	99.5
	150	300	251	3.49	27.9	12.5	2.67	Crimped	0.75	1050	1.00	0.70	85	63.75	82.2
	150	300	251	3.49	34.7	12.5	2.67	Crimped	0.75	1025	1.00	0.60	50	37.50	100.2

	150	300	251	3.49	36.2	12.5	2.67	Crimped	0.75	1025	1.00	0.60	50	37.50	101.0
	150	300	251	3.49	37.0	12.5	2.67	Crimped	0.75	1050	1.00	0.70	85	63.75	111.1
	150	300	251	3.49	38.3	12.5	2.67	Crimped	0.75	1050	1.00	0.70	85	63.75	104.7
Sahoo & Sharma	150	300	261	2.30	28.7	20.0	1.16	Hooked	1.00	1100	0.50	0.75	80	40.00	144.1
	150	300	261	3.45	32.9	20.0	1.95	Hooked	1.00	1100	0.75	0.75	80	60.00	109.5
	150	300	261	3.45	23.8	20.0	1.95	Hooked	1.00	1100	1.00	0.75	80	80.00	94.2
	150	300	261	3.45	24.1	20.0	1.95	Hooked	1.00	1100	1.25	0.75	80	100.00	114.6
Shoaib et al.	310	308	258	3.00	22.0	10.0	1.84	Hooked	1.00	1100	1.00	0.50	55	55.00	203.9
	310	308	258	3.00	31.0	10.0	2.45	Hooked	1.00	1100	1.00	0.50	55	55.00	298.9
	300	600	550	3.00	30.0	10.0	1.19	Hooked	1.00	1100	1.00	0.50	55	55.00	311.6
Manju et al.	140	220	175	1.50	82.0	12.0	1.28	Hooked	1.00	1100	0.50	0.45	80	40.00	118.6
	140	220	175	1.50	83.2	12.0	1.28	Hooked	1.00	1100	1.00	0.45	80	80.00	155.6
	140	220	175	1.50	83.8	12.0	1.28	Hooked	1.00	1100	1.50	0.45	80	120.00	187.2
	140	220	175	2.50	82.0	12.0	1.28	Hooked	1.00	1100	0.50	0.45	80	40.00	63.5
	140	220	175	2.50	83.2	12.0	1.28	Hooked	1.00	1100	1.00	0.45	80	80.00	80.2
	140	220	175	2.50	83.8	12.0	1.28	Hooked	1.00	1100	1.50	0.45	80	120.00	135.8
Arslan et al.	150	230	200	2.50	33.7	22.0	1.34	Hooked	1.00	1100	1.00	0.55	55	54.50	65.4
	150	230	200	2.50	24.5	22.0	1.34	Hooked	1.00	1100	1.00	0.55	55	54.50	44.4
	150	230	200	2.50	21.4	22.0	1.34	Hooked	1.00	1100	2.00	0.55	55	109.00	50.4
	150	230	200	2.50	9.8	12.0	1.34	Hooked	1.00	1100	3.00	0.55	55	163.50	39.4
	150	230	200	3.50	20.2	22.0	1.34	Hooked	1.00	1100	1.00	0.55	55	54.50	33.1
	150	230	200	3.50	21.4	22.0	1.34	Hooked	1.00	1100	2.00	0.55	55	109.00	43.1
	150	230	200	3.50	27.9	12.0	1.34	Hooked	1.00	1100	3.00	0.55	55	163.50	59.1
	150	230	200	4.50	24.5	22.0	1.34	Hooked	1.00	1100	1.00	0.55	55	54.50	43.3
	150	230	200	4.50	21.4	22.0	1.34	Hooked	1.00	1100	2.00	0.55	55	109.00	35.8
Parra-Montesinos et al.	152	457	381	3.40	49.2	10.0	2.71	Hooked	1.00	1100	1.00	0.51	80	80.00	174.4
	152	457	381	3.40	31.0	10.0	2.71	Hooked	1.00	1100	1.50	0.51	60	90.00	150.7
	152	457	381	3.40	44.9	10.0	2.71	Hooked	1.00	1100	1.50	0.51	60	90.00	191.2
	152	457	381	3.40	44.9	10.0	2.71	Hooked	1.00	1100	1.50	0.51	60	90.00	192.4
	152	457	381	3.40	49.2	10.0	2.71	Hooked	1.00	1100	1.00	0.51	80	80.00	220.2
	152	457	381	3.40	31.0	10.0	2.71	Hooked	1.00	1100	1.50	0.51	60	90.00	197.6
	152	457	381	3.50	38.1	10.0	2.71	Hooked	1.00	1100	1.00	0.51	60	60.00	149.0
	152	457	381	3.50	38.1	10.0	2.71	Hooked	1.00	1100	1.00	0.51	60	60.00	202.8

	152	457	381	3.50	38.1	10.0	1.97	Hooked	1.00	1100	1.00	0.51	60	60.00	177.9
	152	457	381	3.50	38.1	10.0	1.97	Hooked	1.00	1100	1.00	0.51	60	60.00	181.4
	200	300	260	1.50	41.2	10.0	1.81	Hooked	1.00	1100	0.25	0.89	67	16.75	279.8
	200	300	260	1.50	40.3	10.0	1.81	Hooked	1.00	1100	0.76	0.89	67	50.92	299.5
	200	300	260	2.50	40.0	10.0	1.81	Hooked	1.00	1100	0.25	0.89	67	16.75	107.6
	200	300	260	2.50	38.7	10.0	1.81	Hooked	1.00	1100	0.76	0.89	67	50.92	144.0
	200	300	260	2.50	40.0	10.0	1.15	Hooked	1.00	1100	0.25	0.89	67	16.75	81.6
	200	300	260	2.50	38.7	10.0	1.15	Hooked	1.00	1100	0.76	0.89	67	50.92	107.1
	200	500	460	3.40	37.7	10.0	2.80	Hooked	1.00	1100	0.50	0.89	67	33.50	243.8
	200	500	460	3.40	38.8	10.0	2.80	Hooked	1.00	1100	0.50	0.89	67	33.50	252.1
	200	500	460	3.40	37.7	10.0	2.80	Hooked	1.00	1100	0.50	0.89	67	33.50	258.5
Rosenbusch & Teutsch	200	500	460	3.40	37.7	10.0	2.80	Hooked	1.00	1100	0.50	0.89	67	33.50	263.1
	200	300	260	3.50	46.9	10.0	3.56	Hooked	1.00	1100	0.25	0.89	67	16.75	109.7
	200	300	260	3.50	43.7	10.0	3.56	Hooked	1.00	1100	0.51	0.89	67	34.17	120.1
	200	300	260	3.50	48.3	10.0	3.56	Hooked	1.00	1100	0.76	0.89	67	50.92	155.0
	200	300	260	3.50	37.7	10.0	2.83	Hooked	1.00	1100	0.50	0.89	67	33.50	110.8
	200	300	260	3.50	38.8	10.0	2.83	Hooked	1.00	1100	0.50	0.89	67	33.50	131.6
	200	600	540	3.50	37.7	10.0	2.73	Hooked	1.00	1100	0.25	0.89	67	16.75	153.4
	200	600	560	3.50	38.8	10.0	2.73	Hooked	1.00	1100	0.50	0.89	67	33.50	229.6
	200	300	260	4.00	41.2	10.0	1.81	Hooked	1.00	1100	0.25	0.89	67	16.75	82.2
	200	300	260	4.00	40.3	10.0	1.81	Hooked	1.00	1100	0.76	0.89	67	50.92	117.0
	150	250	217	1.59	35.0	10.0	1.85	Hooked	1.00	1100	0.75	0.75	80	60.00	148.5
Sahoo et al.	150	250	217	2.47	35.0	10.0	1.85	Hooked	1.00	1100	0.75	0.75	80	60.00	98.8
	150	250	217	2.95	35.0	10.0	1.85	Hooked	1.00	1100	0.75	0.75	80	60.00	84.8
Amin & Foster	300	700	622	2.81	34.0	10.0	1.98	Hooked	1.00	2300	0.32	0.90	65	20.87	285.8
	300	700	622	2.81	36.0	10.0	1.98	Hooked	1.00	2300	0.69	0.90	65	44.66	355.8
	100	150	135	2.22	64.2	15.0	1.16	Hooked	1.00	1100	0.50	0.54	65	32.50	42.2
	100	150	135	2.22	64.2	15.0	1.16	Hooked	1.00	1100	0.50	0.54	65	32.50	44.5
	100	150	135	2.22	64.2	15.0	1.16	Hooked	1.00	1100	0.50	0.54	65	32.50	43.2
Tahenni et al.	100	150	135	2.22	64.0	15.0	1.16	Hooked	1.00	1100	1.00	0.54	65	65.00	45.1
	100	150	135	2.22	64.0	15.0	1.16	Hooked	1.00	1100	1.00	0.54	65	65.00	47.8
	100	150	135	2.22	64.0	15.0	1.16	Hooked	1.00	1100	1.00	0.54	65	65.00	43.0
	100	150	135	2.22	60.0	15.0	1.16	Hooked	1.00	1100	1.00	0.75	80	80.00	49.5

	100	150	135	2.22	60.0	15.0	1.16	Hooked	1.00	1100	1.00	0.75	80	80.00	52.4
	100	150	135	2.22	60.0	15.0	1.16	Hooked	1.00	1100	1.00	0.75	80	80.00	45.2
Narayanan&Darwish	85	150	130	2.02	51.9	9.6	2.05	Crimped	0.75	2000	0.25	0.30	100	18.75	32.9
	85	150	130	2.52	51.9	9.6	2.05	Crimped	0.75	2000	0.25	0.30	100	18.75	29.7
	85	150	130	3.02	51.9	9.6	2.05	Crimped	0.75	2000	0.25	0.30	100	18.75	30.8
	85	150	130	2.02	33.3	9.6	2.05	Crimped	0.75	2000	0.25	0.30	100	18.75	30.1
	85	150	130	2.52	33.3	9.6	2.05	Crimped	0.75	2000	0.25	0.30	100	18.75	23.0
	85	150	130	3.02	33.3	9.6	2.05	Crimped	0.75	2000	0.25	0.30	100	18.75	21.6
	85	150	130	3.02	51.7	9.6	2.05	Crimped	0.75	2000	0.50	0.30	133	49.88	35.9
	85	150	130	3.02	30.6	9.6	2.05	Crimped	0.75	2000	0.50	0.30	133	49.88	22.0
	85	150	130	3.02	31.0	9.6	2.05	Crimped	0.75	2000	1.00	0.30	100	75.00	33.0
	85	150	130	2.02	51.7	9.6	2.05	Crimped	0.75	2000	0.50	0.30	133	49.88	51.2
	85	150	130	2.52	51.7	9.6	2.05	Crimped	0.75	2000	0.50	0.30	133	49.88	40.9
	85	150	130	3.52	41.7	9.6	2.05	Crimped	0.75	2000	0.50	0.30	133	49.88	29.0
	85	150	130	2.02	48.7	9.6	2.05	Crimped	0.75	2000	1.00	0.30	133	99.75	61.7
	85	150	130	2.52	48.7	9.6	2.05	Crimped	0.75	2000	1.00	0.30	133	99.75	49.0
	85	150	130	3.52	48.8	9.6	2.05	Crimped	0.75	2000	1.00	0.30	133	99.75	33.0
	85	150	128	3.06	41.7	9.6	3.70	Crimped	0.75	2000	0.50	0.30	133	49.88	32.4
	85	150	126	3.11	41.7	9.6	5.72	Crimped	0.75	2000	0.50	0.30	133	49.88	38.2
	85	150	128	3.06	30.6	9.6	3.70	Crimped	0.75	2000	0.50	0.30	133	49.88	24.6
	85	150	126	3.11	30.6	9.6	5.72	Crimped	0.75	2000	0.50	0.30	133	49.88	25.1
	85	150	128	3.06	48.8	9.6	3.70	Crimped	0.75	2000	1.00	0.30	133	99.75	47.7
	85	150	126	3.11	48.8	9.6	5.72	Crimped	0.75	2000	1.00	0.30	133	99.75	53.7
	85	150	126	3.11	53.6	9.6	5.72	Crimped	0.75	2000	1.50	0.30	100	112.50	52.1
	85	150	126	3.11	43.2	9.6	5.72	Crimped	0.75	2000	2.00	0.30	100	150.00	53.0
	85	150	128	3.06	53.6	9.6	3.70	Crimped	0.75	2000	1.50	0.30	100	112.50	48.7
	85	150	126	2.08	50.2	9.6	5.72	Crimped	0.75	2000	0.50	0.30	100	37.50	58.6
	85	150	126	2.08	45.9	9.6	5.72	Crimped	0.75	2000	1.00	0.30	100	75.00	72.7
	85	150	126	2.08	53.6	9.6	5.72	Crimped	0.75	2000	1.50	0.30	100	112.50	76.7
	85	150	126	2.08	43.2	9.6	5.72	Crimped	0.75	2000	2.00	0.30	100	150.00	67.6
Cucchiara et al.	150	250	219	2.80	40.9	10.0	1.91	Hooked	1.00	1115	1.00	0.50	60	60.00	97.5
	150	250	219	2.80	40.9	10.0	1.91	Hooked	1.00	1115	2.00	0.50	60	120.00	104.4
	150	250	219	2.00	43.2	10.0	1.91	Hooked	1.00	1115	1.00	0.50	60	60.00	116.2

	150	250	219	2.00	43.2	10.0	1.91	Hooked	1.00	1115	2.00	0.50	60	120.00	116.6
Kwak et al.	125	250	212	2.00	63.8	19.0	1.52	Hooked	1.00	1079	0.50	0.80	63	31.25	135.4
	125	250	212	2.00	68.6	19.0	1.52	Hooked	1.00	1079	0.75	0.80	63	46.88	144.6
	125	250	212	2.00	30.8	19.0	1.52	Hooked	1.00	1079	0.50	0.80	63	31.25	107.5
	125	250	212	3.00	30.8	19.0	1.52	Hooked	1.00	1079	0.50	0.80	63	31.25	68.2
Lim & Oh	100	180	130	3.08	38.7	10.0	3.09	Straight smooth	0.50	1303	1.00	0.70	60	30.00	58.7
	100	180	130	3.08	42.4	10.0	3.09	Straight smooth	0.50	1303	2.00	0.70	60	60.00	74.8
Dinh et al.	152	455	381	3.44	44.8	10.0	1.96	Hooked	1.00	1100	0.75	0.54	55	41.25	172.4
	152	455	381	3.44	44.8	10.0	1.96	Hooked	1.00	1100	0.75	0.54	55	41.25	161.6
	152	455	381	3.44	38.1	10.0	1.96	Hooked	1.00	1100	1.00	0.54	55	55.00	170.8
	152	455	381	3.44	38.1	10.0	1.96	Hooked	1.00	1100	1.00	0.54	55	55.00	173.9
	152	455	381	3.44	31.0	10.0	2.63	Hooked	1.00	1100	1.50	0.54	55	82.50	150.3
	152	455	381	3.44	31.0	10.0	2.63	Hooked	1.00	1100	1.50	0.54	55	82.50	197.9
	152	455	381	3.44	44.9	10.0	2.63	Hooked	1.00	1100	1.50	0.54	55	82.50	192.9
	152	455	381	3.44	44.9	10.0	2.63	Hooked	1.00	1100	1.50	0.54	55	82.50	191.3
	152	455	381	3.44	49.2	10.0	2.63	Hooked	1.00	1100	1.00	0.75	80	80.00	173.9
	152	455	381	3.44	49.2	10.0	2.63	Hooked	1.00	1100	1.00	0.75	80	80.00	220.3
	152	455	381	3.44	43.3	10.0	1.96	Hooked	1.00	2300	0.75	0.38	80	60.00	194.4
	152	455	381	3.44	43.3	10.0	1.96	Hooked	1.00	2300	0.75	0.38	80	60.00	191.3
	205	685	610	3.50	50.8	10.0	1.96	Hooked	1.00	1100	0.75	0.54	55	41.25	369.1
	205	685	610	3.50	50.8	10.0	1.96	Hooked	1.00	1100	0.75	0.54	55	41.25	340.8
	205	685	610	3.50	28.7	10.0	1.96	Hooked	1.00	1100	0.75	0.75	80	60.00	354.8
	205	685	610	3.50	28.7	10.0	1.96	Hooked	1.00	1100	0.75	0.75	80	60.00	347.6
	205	685	610	3.50	42.3	10.0	1.52	Hooked	1.00	1100	0.75	0.54	55	41.25	351.2
	205	685	610	3.50	29.6	10.0	1.52	Hooked	1.00	1100	0.75	0.75	80	60.00	271.2
	205	685	610	3.50	29.6	10.0	1.52	Hooked	1.00	1100	0.75	0.75	80	60.00	228.5
	205	685	610	3.50	44.4	10.0	1.96	Hooked	1.00	1100	1.50	0.54	55	82.50	438.3
	205	685	610	3.50	42.8	10.0	1.96	Hooked	1.00	1100	1.50	0.75	80	120.00	424.3
Lima Araujo et al.	150	390	340	2.50	58.9	12.5	3.08	Hooked	1.00	1150	1.00	0.54	65	65.00	261.6
	150	390	340	2.50	51.7	12.5	3.08	Hooked	1.00	1150	2.00	0.54	65	130.00	292.1
Casanova et al.	150	800	735	3.81	42.0	12.5	1.06	Hooked	1.00	1200	1.25	0.80	75	93.75	359.9
	150	800	735	3.81	38.0	12.5	1.06	Hooked	1.00	1200	1.25	0.50	60	75.00	359.9
	125	250	225	2.89	90.0	10.0	3.49	Hooked	1.00	1200	1.25	0.50	60	75.00	157.8

Aoude et al.	150	250	202	2.97	21.3	10.0	1.17	Hooked	1.00	1100	0.50	0.55	55	27.50	48.3
	150	250	202	2.97	19.6	10.0	1.17	Hooked	1.00	1100	1.00	0.55	55	55.00	57.3
	300	500	437	3.09	21.3	10.0	1.50	Hooked	1.00	1100	0.50	0.55	55	27.50	161.2
	300	500	437	3.09	19.6	10.0	1.50	Hooked	1.00	1100	1.00	0.55	55	55.00	204.9
Minelli & Plizzari	200	480	435	2.51	24.8	20.0	1.04	Hooked	1.00	1100	0.38	0.60	50	19.00	134.2
	200	480	435	2.51	33.5	20.0	1.04	Hooked	1.00	1100	0.38	1.00	50	19.00	119.7
	200	480	435	2.51	33.5	20.0	1.04	Hooked+straight	0.75	1333	0.57	0.73	78	33.35	141.7
	200	480	435	2.51	38.6	20.0	1.04	Hooked	1.00	1100	0.38	0.60	50	19.00	141.2
	200	480	435	2.51	61.1	20.0	1.04	Hooked	1.00	1250	0.64	0.62	48	30.72	191.2
	200	500	455	2.51	24.4	15.0	0.99	Hooked	1.00	1100	0.25	1.00	50	12.50	196.9
	200	500	455	2.51	24.4	15.0	0.99	Hooked	1.00	1100	0.25	1.00	50	12.50	156.9
	200	1000	910	2.50	24.4	20.0	1.04	Hooked	1.00	1100	0.25	1.00	50	12.50	258.4
	200	1000	910	2.50	55.0	20.0	1.04	Hooked	1.00	1100	0.25	1.00	50	12.50	339.4
Kang et al.	125	250	210	2.00	44.6	19.0	1.53	Hooked	1.00	1100	0.50	0.80	63	31.25	82.1
	125	250	210	4.00	44.6	19.0	1.53	Hooked	1.00	1100	0.50	0.80	63	31.25	36.0
	125	250	210	2.00	57.2	19.0	1.53	Hooked	1.00	1100	0.50	0.80	63	31.25	77.7
Casanova & Rossi	125	250	225	2.89	90.0	10.0	3.49	Hooked	1.00	1200	1.25	0.50	60	75.00	138.8
	125	250	225	2.89	90.0	10.0	3.49	Hooked	1.00	1200	1.25	0.50	60	75.00	138.8
Lim et al.	152	254	221	2.50	34.0	10.0	1.20	Hooked	1.00	1130	0.50	0.50	60	30.00	59.0
	152	254	221	1.50	34.0	10.0	2.39	Hooked	1.00	1130	1.00	0.50	60	60.00	148.3
	152	254	221	2.50	34.0	10.0	2.39	Hooked	1.00	1130	1.00	0.50	60	60.00	83.6
	152	254	221	3.50	34.0	10.0	2.39	Hooked	1.00	1130	1.00	0.50	60	60.00	68.4
	152	254	221	1.50	34.0	10.0	2.39	Hooked	1.00	1130	0.50	0.50	60	30.00	135.8
	152	254	221	2.50	34.0	10.0	2.39	Hooked	1.00	1130	0.50	0.50	60	30.00	64.7
	152	254	221	3.50	34.0	10.0	2.39	Hooked	1.00	1130	0.50	0.50	60	30.00	50.4
Mansur et al.	150	225	197	2.00	29.1	20.0	1.36	Hooked	1.00	1260	0.50	0.50	60	30.00	75.5
	150	225	197	2.80	29.1	20.0	1.36	Hooked	1.00	1260	0.50	0.50	60	30.00	53.2
	150	225	197	3.60	29.1	20.0	1.36	Hooked	1.00	1260	0.50	0.50	60	30.00	45.8
	150	225	197	2.00	29.9	20.0	1.36	Hooked	1.00	1260	0.75	0.50	60	45.00	85.5
	150	225	197	2.80	29.9	20.0	1.36	Hooked	1.00	1260	0.75	0.50	60	45.00	60.7
	150	225	197	2.80	29.9	20.0	2.04	Hooked	1.00	1260	0.75	0.50	60	45.00	65.7
	150	225	197	2.80	20.6	20.0	1.36	Hooked	1.00	1260	0.75	0.50	60	45.00	45.7
	150	225	197	2.80	20.6	20.0	2.04	Hooked	1.00	1260	0.75	0.50	60	45.00	60.7

	150	225	197	2.80	33.4	20.0	2.04	Hooked	1.00	1260	0.75	0.50	60	45.00	86.7
Zarrinpour & Chao	152	305	254	3.50	29.0	10.0	2.48	Hooked	1.00	1096	0.75	0.76	67	50.25	121.0
	610	305	254	3.50	29.0	10.0	2.47	Hooked	1.00	1096	0.75	0.76	67	50.25	482.1
	152	457	394	3.61	39.0	10.0	2.86	Hooked	1.00	1096	0.75	0.76	67	50.25	163.5
	152	457	394	3.61	39.0	10.0	2.86	Hooked	1.00	1096	0.75	0.76	67	50.25	196.5
	203	610	541	3.45	50.0	10.0	2.54	Hooked	1.00	1096	0.75	0.76	67	50.25	272.8
	203	610	541	3.45	50.0	10.0	2.54	Hooked	1.00	1096	0.75	0.76	67	50.25	385.8
	254	915	813	3.50	50.0	10.0	2.70	Hooked	1.00	1096	0.75	0.76	67	50.25	699.5
	254	915	813	3.50	50.0	10.0	2.70	Hooked	1.00	1096	0.75	0.76	67	50.25	720.5
	305	1220	1118	3.50	50.0	10.0	2.55	Hooked	1.00	1096	0.75	0.76	67	50.25	1081.4
	305	1220	1118	3.50	50.0	10.0	2.55	Hooked	1.00	1096	0.75	0.76	67	50.25	1044.4
Noghabai	200	250	180	3.33	90.6	16.0	4.47	Straight smooth	0.50	2600	1.00	0.15	40	20.00	299.8
	200	250	180	3.33	83.2	16.0	4.47	Hooked+straight	0.75	1850	1.00	0.38	48	36.00	295.8
	200	250	180	3.33	80.5	16.0	4.47	Hooked	1.00	2200	0.50	0.70	86	42.86	252.8
	200	250	180	3.33	80.5	16.0	4.47	Hooked	1.00	2200	0.75	0.70	86	64.29	262.8
	200	250	195	3.08	39.4	16.0	3.09	Hooked+straight	0.75	1850	1.00	0.38	48	36.00	189.8
	200	300	235	2.77	91.4	16.0	4.28	Hooked	1.00	1100	1.00	0.60	50	50.00	311.0
	200	300	235	2.77	93.3	16.0	4.28	Straight smooth	0.50	2600	1.00	0.15	40	20.00	364.0
	200	300	235	2.77	89.6	16.0	4.28	Hooked+straight	0.75	1850	1.00	0.38	48	36.00	408.0
	200	500	410	2.93	76.8	18.0	3.06	Straight smooth	0.50	2600	1.00	0.15	40	20.00	292.8
	200	500	410	2.93	76.8	18.0	3.06	Straight smooth	0.50	2600	1.00	0.15	40	20.00	339.8
	200	500	410	2.93	72.0	18.0	3.06	Hooked+straight	0.75	1850	1.00	0.38	48	36.00	370.8
	200	500	410	2.93	72.0	18.0	3.06	Hooked+straight	0.75	1850	1.00	0.38	48	36.00	330.8
	200	500	410	2.93	69.3	18.0	3.06	Hooked	1.00	2200	0.50	0.70	86	42.86	267.8
	200	500	410	2.93	69.3	18.0	3.06	Hooked	1.00	2200	0.50	0.70	86	42.86	315.8
	200	500	410	2.93	60.2	18.0	3.06	Hooked	1.00	2200	0.75	0.70	86	64.29	342.8
	200	500	410	2.93	75.7	18.0	3.06	Hooked	1.00	2200	0.75	0.70	86	64.29	295.8
	300	700	570	2.98	76.8	18.0	2.87	Straight smooth	0.50	2600	1.00	0.15	40	20.00	458.1
	300	700	570	2.98	72.0	18.0	2.87	Hooked+straight	0.75	1850	1.00	0.38	48	36.00	609.1
	300	700	570	2.98	60.2	18.0	2.87	Hooked	1.00	2200	0.75	0.70	86	64.29	522.1
Randl et al.	200	350	314	3.50	131.5	0.4	3.50	Straight smooth	0.50	2000	2.00	0.20	75	75.00	253.6
	200	350	314	3.50	154.5	0.4	3.50	Straight smooth	0.50	2000	2.00	0.20	75	75.00	320.6
	200	350	314	3.50	145.6	0.4	3.50	Straight smooth	0.50	2000	2.00	0.20	75	75.00	359.6

	200	350	314	3.50	132.8	0.4	3.50	Straight smooth	0.50	2000	1.00	0.20	75	37.50	268.6
	200	350	314	3.50	143.3	0.4	3.50	Straight smooth	0.50	2000	1.00	0.20	75	37.50	201.6
	200	350	314	3.50	152.9	0.4	3.50	Straight smooth	0.50	2000	1.00	0.20	75	37.50	310.6
Ashour et al.	125	250	215	2.00	92.0	10.0	0.37	Hooked	1.00	260	1.00	0.80	75	75.00	45.7
	125	250	215	4.00	92.6	10.0	0.37	Hooked	1.00	260	1.00	0.80	75	75.00	24.9
	125	250	215	6.00	93.7	10.0	0.37	Hooked	1.00	260	1.00	0.80	75	75.00	16.3
	125	250	215	1.00	99.0	10.0	2.83	Hooked	1.00	260	0.50	0.80	75	37.50	244.7
	125	250	215	2.00	99.1	10.0	2.83	Hooked	1.00	260	0.50	0.80	75	37.50	130.1
	125	250	215	4.00	95.4	10.0	2.83	Hooked	1.00	260	0.50	0.80	75	37.50	61.9
	125	250	215	6.00	95.8	10.0	2.83	Hooked	1.00	260	0.50	0.80	75	37.50	53.6
	125	250	215	1.00	95.3	10.0	2.83	Hooked	1.00	260	1.00	0.80	75	75.00	342.8
	125	250	215	2.00	95.3	10.0	2.83	Hooked	1.00	260	1.00	0.80	75	75.00	163.4
	125	250	215	4.00	97.5	10.0	2.83	Hooked	1.00	260	1.00	0.80	75	75.00	86.1
	125	250	215	6.00	100.5	10.0	2.83	Hooked	1.00	260	1.00	0.80	75	75.00	53.9
	125	250	215	1.00	96.4	10.0	2.83	Hooked	1.00	260	1.50	0.80	75	112.50	375.3
	125	250	215	2.00	96.6	10.0	2.83	Hooked	1.00	260	1.50	0.80	75	112.50	194.3
	125	250	215	4.00	97.1	10.0	2.83	Hooked	1.00	260	1.50	0.80	75	112.50	95.2
	125	250	215	6.00	101.3	10.0	2.83	Hooked	1.00	260	1.50	0.80	75	112.50	54.4
	125	250	215	2.00	94.5	10.0	4.58	Hooked	1.00	260	1.00	0.80	75	75.00	181.4
	125	250	215	4.00	93.8	10.0	4.58	Hooked	1.00	260	1.00	0.80	75	75.00	105.1
	125	250	215	6.00	95.0	10.0	4.58	Hooked	1.00	260	1.00	0.80	75	75.00	79.9
Tan et al.	140	375	340	2.00	35.0	19.0	1.67	Hooked	1.00	1100	0.50	0.50	60	30.00	219.3
	140	375	340	2.00	33.0	19.0	1.67	Hooked	1.00	1100	0.75	0.50	60	45.00	182.2
	140	375	340	2.00	36.0	19.0	1.67	Hooked	1.00	1100	1.00	0.50	60	60.00	211.6
	140	375	340	2.50	36.0	19.0	1.67	Hooked	1.00	1100	1.00	0.50	60	60.00	155.5
	140	375	340	1.50	36.0	19.0	1.67	Hooked	1.00	1100	1.00	0.50	60	60.00	308.3
Pansuk et al.	150	400	350	2.86	121.1	2.0	5.61	Hooked	1.00	2000	0.80	0.20	65	52.00	341.5
	150	400	350	2.86	120.3	2.0	5.61	Hooked	1.00	2000	1.60	0.20	65	104.00	532.5
Kim et al.	260	400	340	4.00	21.0	10.0	1.72	Hooked	1.00	1336	0.75	0.50	60	45.00	118.1
	260	400	340	4.00	56.0	10.0	1.72	Hooked	1.00	1336	0.75	0.50	60	45.00	208.1
Sharma	150	300	276	1.81	48.6	10.0	1.46	Hooked	1.00	1100	0.96	0.60	85	81.60	123.5
Narayanan&Darwish	100	400	345	0.70	52.9	5.0	3.55	Crimped	0.75	2000	0.25	0.30	100	18.75	350.5
	100	400	345	0.70	51.0	5.0	3.55	Crimped	0.75	2000	0.50	0.30	100	37.50	325.5

	100	400	345	0.70	47.6	5.0	3.55	Crimped	0.75	2000	0.75	0.30	100	56.25	361.5
	100	400	345	0.70	55.9	5.0	3.55	Crimped	0.75	2000	1.00	0.30	100	75.00	396.5
	100	400	345	0.70	54.9	5.0	3.55	Crimped	0.75	2000	1.25	0.30	100	93.75	393.5
	100	400	345	0.46	50.5	5.0	3.55	Crimped	0.75	2000	1.00	0.30	100	75.00	454.5
	100	400	345	0.58	47.8	5.0	3.55	Crimped	0.75	2000	1.00	0.30	100	75.00	404.5
	100	400	345	0.81	45.6	5.0	3.55	Crimped	0.75	2000	1.00	0.30	100	75.00	342.5
	100	400	345	0.93	49.1	5.0	3.55	Crimped	0.75	2000	1.00	0.30	100	75.00	344.5
	100	400	345	0.70	31.0	5.0	3.55	Crimped	0.75	2000	1.00	0.30	100	75.00	294.5
	100	400	345	0.70	34.7	5.0	3.55	Crimped	0.75	2000	1.00	0.30	100	75.00	333.5
Li et al.	64	127	102	3.00	53.0	2.4	2.20	Crimped	0.75	1000	1.00	0.88	29	21.38	16.6
	127	228	204	3.00	53.0	2.4	2.21	Crimped	0.75	1000	1.00	0.88	29	21.38	51.0
	64	127	102	3.00	50.2	2.4	2.20	Crimped	0.75	1000	2.00	0.88	29	42.75	20.9
	127	228	204	3.00	50.2	2.4	2.21	Crimped	0.75	1000	2.00	0.88	29	42.75	66.8
	64	127	102	3.00	62.6	2.4	2.20	Crimped	0.75	1000	1.00	0.88	29	21.38	17.8
	127	228	204	3.00	62.6	2.4	2.21	Crimped	0.75	1000	1.00	0.88	29	21.38	61.8
	64	127	102	1.00	62.6	2.4	2.20	Crimped	0.75	1000	1.00	0.88	29	21.38	50.7
	64	127	102	1.50	62.6	2.4	2.20	Crimped	0.75	1000	1.00	0.88	29	21.38	33.4
	64	127	102	1.75	62.6	2.4	2.20	Crimped	0.75	1000	1.00	0.88	29	21.38	30.1
	64	127	102	2.00	62.6	2.4	2.20	Crimped	0.75	1000	1.00	0.88	29	21.38	25.9
	64	127	102	2.25	62.6	2.4	2.20	Crimped	0.75	1000	1.00	0.88	29	21.38	23.5
	64	127	102	2.50	62.6	2.4	2.20	Crimped	0.75	1000	1.00	0.88	29	21.38	20.6
	64	127	102	2.75	62.6	2.4	2.20	Crimped	0.75	1000	1.00	0.88	29	21.38	17.9
	64	127	102	3.00	62.6	2.4	1.10	Crimped	0.75	1000	1.00	0.88	29	21.38	12.9
	64	127	102	3.00	62.6	2.4	3.30	Crimped	0.75	1000	1.00	0.88	29	21.38	17.9
	64	127	102	3.00	54.1	2.4	3.30	Crimped	0.75	1000	1.00	0.88	57	42.75	25.3
	127	228	204	3.00	22.7	9.0	2.21	Hooked	1.00	1172	1.00	0.50	60	60.00	79.5
	64	127	102	3.00	22.7	9.0	2.20	Hooked	1.00	1172	1.00	0.50	60	60.00	20.5
	64	127	102	3.00	22.7	9.0	1.10	Hooked	1.00	1172	1.00	0.50	60	60.00	15.8
	64	127	102	1.50	22.7	9.0	1.10	Hooked	1.00	1172	1.00	0.50	60	60.00	36.6
	127	228	204	3.00	26.0	9.0	2.21	Hooked	1.00	1172	1.00	0.50	100	100.00	79.5
	64	127	102	3.00	26.0	9.0	2.20	Hooked	1.00	1172	1.00	0.50	100	100.00	23.1
Swamy et al.	55	300	265	2.00	36.5	14.0	4.31	Crimped	0.75	1570	1.00	0.50	100	75.00	80.7
	55	300	265	3.43	41.9	14.0	4.31	Crimped	0.75	1570	1.00	0.50	100	75.00	59.4

	55	300	265	4.91	36.9	14.0	4.31	Crimped	0.75	1570	1.00	0.50	100	75.00	42.9
	55	300	265	2.00	38.7	14.0	2.76	Crimped	0.75	1570	1.00	0.50	100	75.00	72.3
	55	300	265	3.43	33.9	14.0	2.76	Crimped	0.75	1570	1.00	0.50	100	75.00	46.0
	55	300	265	4.91	36.8	14.0	2.76	Crimped	0.75	1570	1.00	0.50	100	75.00	43.2
	55	300	265	2.00	36.6	14.0	1.55	Crimped	0.75	1570	1.00	0.50	100	75.00	68.2
Adebar et al.	150	610	560	1.63	54.1	14.0	2.14	Hooked	1.00	1200	0.75	0.50	60	45.00	277.7
	150	610	560	1.63	49.9	14.0	2.14	Hooked	1.00	1200	1.50	0.50	60	90.00	325.7
	150	610	560	1.63	54.8	14.0	2.14	Hooked	1.00	1200	0.40	0.50	60	24.00	205.7
	150	610	560	1.63	56.5	14.0	2.14	Hooked	1.00	1200	0.60	0.50	60	36.00	233.7
	150	610	560	1.63	46.9	14.0	2.14	Hooked	1.00	1200	0.40	0.50	60	24.00	248.7
	150	610	560	1.63	40.8	14.0	2.14	Hooked	1.00	1200	0.60	0.50	60	36.00	238.7
Cho & Kim	120	200	168	1.43	25.7	13.0	1.32	Hooked	1.00	1100	0.50	0.60	60	30.00	61.1
	120	200	168	1.43	25.3	13.0	1.32	Hooked	1.00	1100	1.00	0.60	60	60.00	79.6
	120	200	168	1.43	23.9	13.0	1.32	Hooked	1.00	1100	1.50	0.60	60	90.00	84.5
	120	200	168	1.43	57.8	13.0	1.32	Hooked	1.00	1100	0.50	0.60	60	30.00	95.4
	120	200	168	1.43	61.5	13.0	1.32	Hooked	1.00	1100	1.00	0.60	60	60.00	103.2
	120	200	168	1.43	70.5	13.0	2.82	Hooked	1.00	1100	0.50	0.60	60	30.00	179.0
	120	200	168	1.43	67.3	13.0	2.82	Hooked	1.00	1100	1.00	0.60	60	60.00	169.7
	120	200	168	1.43	67.3	13.0	2.82	Hooked	1.00	1100	1.50	0.60	60	90.00	186.9
	120	200	168	1.43	82.4	13.0	2.00	Hooked	1.00	1100	0.50	0.60	60	30.00	158.1
	120	200	168	1.43	81.1	13.0	2.00	Hooked	1.00	1100	1.00	0.60	60	60.00	163.0
	120	200	168	1.43	86.1	13.0	2.82	Hooked	1.00	1100	0.50	0.60	60	30.00	153.7
	120	200	168	1.43	89.4	13.0	2.82	Hooked	1.00	1100	1.00	0.60	60	60.00	170.8
Greenough & Nehdi	200	300	265	3.02	47.9	10.0	1.78	Hooked	1.00	1100	0.50	1.00	50	25.00	92.4
	200	300	265	3.02	38.0	10.0	1.78	Hooked	1.00	1100	0.75	1.00	50	37.50	107.1
	200	300	265	3.02	42.2	10.0	1.78	Hooked	1.00	1100	1.00	1.00	50	50.00	150.4
	200	300	265	3.02	45.4	10.0	1.78	Flat end	0.50	1100	0.50	1.00	50	12.50	117.0
	200	300	265	3.02	44.4	10.0	1.78	Flat end	0.50	1100	0.75	1.00	50	18.75	145.6
	200	300	265	3.02	40.3	10.0	1.78	Flat end	0.50	1100	1.00	1.00	50	25.00	148.3
	200	300	265	3.02	53.7	10.0	1.78	Flat end	0.50	1100	0.50	0.70	43	10.71	108.2
	200	300	265	3.02	46.0	10.0	1.78	Flat end	0.50	1100	0.75	0.70	43	16.07	124.4
	200	300	265	3.02	42.2	10.0	1.78	Flat end	0.50	1100	1.00	0.70	43	21.43	152.9
Kang et al.	200	355	310	2.55	39.8	9.5	1.13	Hooked	1.00	1100	0.38	0.75	80	30.00	133.8

	200	355	285	2.77	39.8	9.5	3.33	Hooked	1.00	1100	0.38	0.75	80	30.00	222.9
	200	300	260	3.46	46.4	14.0	3.55	Hooked	1.00	1100	0.25	0.92	65	16.25	111.4
	200	300	260	3.46	43.2	14.0	3.55	Hooked	1.00	1100	0.50	0.92	65	32.50	121.4
	200	300	260	3.46	47.6	14.0	3.55	Hooked	1.00	1100	0.75	0.92	65	48.75	156.4
	200	300	260	1.54	40.7	14.0	1.81	Hooked	1.00	1100	0.25	0.92	65	16.25	281.7
	200	300	260	1.54	42.4	14.0	1.81	Hooked	1.00	1100	0.75	0.92	65	48.75	301.7
	200	300	262	2.48	39.1	14.0	1.15	Hooked	1.00	1100	0.25	0.92	65	16.25	84.2
	200	300	262	2.48	38.6	14.0	1.15	Hooked	1.00	1100	0.75	0.92	65	48.75	109.7
	200	300	260	2.50	39.1	14.0	1.81	Hooked	1.00	1100	0.25	0.92	65	16.25	109.7
	200	300	260	2.50	38.6	14.0	1.81	Hooked	1.00	1100	0.75	0.92	65	48.75	145.7
Dupont & Vewalle	200	300	260	4.04	40.7	14.0	1.81	Hooked	1.00	1100	0.25	0.92	65	16.25	84.2
	200	300	260	4.04	42.4	14.0	1.81	Hooked	1.00	1100	0.75	0.92	65	48.75	118.7
	200	300	262	2.48	26.5	14.0	1.15	Hooked	1.00	1100	0.25	1.11	45	11.25	101.7
	200	300	262	2.48	27.2	14.0	1.15	Hooked	1.00	1100	0.75	1.11	45	33.75	121.7
	200	300	260	2.50	26.5	14.0	1.81	Hooked	1.00	1100	0.25	1.11	45	11.25	101.7
	200	300	260	2.50	27.2	14.0	1.81	Hooked	1.00	1100	0.75	1.11	45	33.75	121.7
	200	300	262	2.48	47.4	14.0	1.15	Hooked	1.00	1100	0.50	0.92	65	32.50	131.7
	200	300	260	2.50	46.8	14.0	1.81	Hooked	1.00	1100	0.50	0.92	65	32.50	159.2
	200	300	262	2.48	45.4	14.0	1.15	Hooked	1.00	1100	0.50	0.63	80	40.00	149.2
	200	350	305	2.46	34.4	14.0	1.03	Hooked	1.00	1100	0.57	0.75	80	45.60	164.8
	200	350	305	2.46	30.2	14.0	1.03	Hooked	1.00	1100	0.38	0.75	80	30.40	164.8
	175	250	210	4.50	36.4	10.0	4.01	Crimped	0.75	1050	0.40	0.50	100	30.00	81.0
	175	250	210	4.50	38.4	10.0	4.01	Crimped	0.75	1050	0.80	0.50	100	60.00	115.5
Swamy & Bahia	175	250	210	4.50	40.8	10.0	4.01	Crimped	0.75	1050	1.20	0.50	100	90.00	116.5
	175	250	210	4.50	39.1	10.0	3.10	Crimped	0.75	1050	0.80	0.50	100	60.00	119.7
	175	250	210	4.50	38.5	10.0	4.01	Crimped	0.75	1050	0.80	0.50	100	60.00	70.5
	101	152	127	4.80	33.2	2.0	3.09	Flat	0.50	1100	0.22	0.25	102	11.18	28.2
	101	152	127	4.80	33.2	2.0	3.09	Flat	0.50	1100	0.22	0.25	102	11.18	27.8
	101	152	127	4.80	33.2	2.0	3.09	Flat	0.50	1100	0.22	0.25	102	11.18	27.2
Batson et al.	101	152	127	4.80	33.2	2.0	3.09	Crimped	0.75	1100	0.22	0.41	46	7.67	28.5
	101	152	127	4.80	33.2	2.0	3.09	Crimped	0.75	1100	0.22	0.41	46	7.67	28.5
	101	152	127	4.80	33.2	2.0	3.09	Crimped	0.75	1100	0.22	0.41	46	7.67	26.7
	101	152	127	4.80	33.2	2.0	3.09	Round	0.50	1100	0.22	0.25	102	11.18	27.1

	101	152	127	4.40	33.2	2.0	3.09	Round	0.50	1100	0.22	0.25	102	11.18	31.9
	101	152	127	4.20	33.2	2.0	3.09	Round	0.50	1100	0.22	0.25	102	11.18	31.8
	101	152	127	4.20	33.2	2.0	3.09	Round	0.50	1100	0.22	0.25	102	11.18	28.2
	101	152	127	4.20	33.2	2.0	3.09	Round	0.50	1100	0.22	0.25	102	11.18	25.5
	101	152	127	4.30	33.2	2.0	3.09	Round	0.50	1100	0.22	0.25	102	11.18	29.9
	101	152	127	4.30	33.2	2.0	3.09	Round	0.50	1100	0.22	0.25	102	11.18	28.3
	101	152	127	4.20	40.2	2.0	3.09	Round	0.50	1100	0.44	0.25	102	22.35	33.3
	101	152	127	4.00	40.2	2.0	3.09	Round	0.50	1100	0.44	0.25	102	22.35	33.4
	101	152	127	4.00	40.2	2.0	3.09	Round	0.50	1100	0.44	0.25	102	22.35	31.5
	101	152	127	4.00	40.2	2.0	3.09	Round	0.50	1100	0.44	0.25	102	22.35	33.4
	101	152	127	4.40	33.2	2.0	3.09	Round	0.50	1100	0.22	0.25	102	11.18	28.7
	101	152	127	4.40	33.2	2.0	3.09	Round	0.50	1100	0.22	0.25	102	11.18	27.2
	101	152	127	4.00	33.2	2.0	3.09	Crimped	0.75	1100	0.22	0.41	62	10.22	30.4
	101	152	127	4.00	33.2	2.0	3.09	Crimped	0.75	1100	0.22	0.41	62	10.22	30.5
	101	152	127	4.00	33.2	2.0	3.09	Crimped	0.75	1100	0.22	0.41	62	10.22	33.4
	101	152	127	4.60	33.2	2.0	3.09	Crimped	0.75	1100	0.22	0.41	62	10.22	26.2
	101	152	127	4.40	33.2	2.0	3.09	Crimped	0.75	1100	0.22	0.41	62	10.22	27.4
	101	152	127	4.40	33.2	2.0	3.09	Crimped	0.75	1100	0.22	0.41	62	10.22	26.0
	101	152	127	5.00	33.2	2.0	3.09	Crimped	0.75	1100	0.22	0.41	62	10.22	24.7
	101	152	127	4.80	33.2	2.0	3.09	Crimped	0.75	1100	0.22	0.41	62	10.22	22.6
	101	152	127	4.00	40.2	2.0	3.09	Crimped	0.75	1100	0.44	0.41	62	20.44	31.8
	101	152	127	4.20	40.2	2.0	3.09	Crimped	0.75	1100	0.44	0.41	62	20.44	34.1
	101	152	127	4.20	40.2	2.0	3.09	Crimped	0.75	1100	0.44	0.41	62	20.44	30.4
	101	152	127	4.20	40.2	2.0	3.09	Crimped	0.75	1100	0.44	0.41	62	20.44	32.8
	101	152	127	3.20	39.7	2.0	3.09	Crimped	0.75	1100	0.88	0.41	62	40.89	37.0
	101	152	127	3.40	39.7	2.0	3.09	Crimped	0.75	1100	0.88	0.41	62	40.89	34.6
	101	152	127	3.40	39.7	2.0	3.09	Crimped	0.75	1100	0.88	0.41	62	40.89	33.4
	101	152	127	3.40	39.7	2.0	3.09	Crimped	0.75	1100	0.88	0.41	62	40.89	42.2
	101	152	127	3.40	39.7	2.0	3.09	Crimped	0.75	1100	0.88	0.41	62	40.89	39.8
	101	152	127	2.80	39.8	2.0	3.09	Crimped	0.75	1100	1.76	0.41	62	81.78	56.5
	101	152	127	1.80	39.8	2.0	3.09	Crimped	0.75	1100	1.76	0.41	62	81.78	77.3
	101	152	127	1.20	39.8	2.0	3.09	Crimped	0.75	1100	1.76	0.41	62	81.78	145.4
	101	152	127	1.20	39.8	2.0	3.09	Crimped	0.75	1100	1.76	0.41	62	81.78	139.7

	101	152	127	4.80	33.2	2.0	3.09	Crimped	0.75	1100	0.22	0.41	62	10.22	24.6
	101	152	127	4.80	33.2	2.0	3.09	Crimped	0.75	1100	0.22	0.41	62	10.22	23.7
	101	152	127	4.80	33.2	2.0	3.09	Crimped	0.75	1100	0.22	0.41	62	10.22	26.4
Zhao et al.	150	300	260	2.00	34.5	20.0	2.52	Mill-cut	0.50	700	0.50	0.92	35	8.75	113.7
	150	300	260	2.00	36.1	20.0	2.52	Mill-cut	0.50	700	1.00	0.92	35	17.50	139.2
	150	300	260	2.00	37.1	20.0	2.52	Mill-cut	0.50	700	1.50	0.92	35	26.25	156.6
	150	300	260	2.00	35.3	20.0	2.52	Mill-cut	0.50	700	2.00	0.92	35	35.00	150.0
Jindal	100	153	127	3.60	20.7	2.0	1.99	Straight mild steel	0.50	4913	1.00	0.28	25	12.50	21.2
	100	153	127	2.00	20.7	2.0	1.99	Brass-coated steel	0.50	2350	1.00	0.25	100	50.00	29.6
	100	153	127	2.40	20.7	2.0	1.99	Brass-coated steel	0.50	2350	1.00	0.25	100	50.00	29.6
	100	153	127	2.00	20.7	2.0	1.99	Brass-coated steel	0.50	2350	1.00	0.15	83	41.67	40.2
	100	153	127	3.60	20.7	2.0	1.99	Brass-coated steel	0.50	2350	1.00	0.15	83	41.67	29.0
	100	153	127	4.80	20.7	2.0	1.99	Brass-coated steel	0.50	2350	1.00	0.15	83	41.67	24.8
	100	153	127	2.00	20.7	2.0	1.99	Brass-coated steel	0.50	2350	1.00	0.40	63	31.25	33.1
Shin et al.	100	200	175	2.00	80.0	13.0	3.59	Smooth straight	0.50	1856	0.50	0.40	100	25.00	119.9
	100	200	175	2.00	80.0	13.0	3.59	Smooth straight	0.50	1856	1.00	0.40	100	50.00	129.7
	100	200	175	3.00	80.0	13.0	3.59	Smooth straight	0.50	1856	0.50	0.40	100	25.00	56.1
	100	200	175	3.00	80.0	13.0	3.59	Smooth straight	0.50	1856	1.00	0.40	100	50.00	72.0
	100	200	175	4.50	80.0	13.0	3.59	Smooth straight	0.50	1856	0.50	0.40	100	25.00	49.0
	100	200	175	4.50	80.0	13.0	3.59	Smooth straight	0.50	1856	1.00	0.40	100	50.00	60.6
Imam et al.	200	350	300	1.75	109.5	10.0	3.08	Hooked	1.00	2000	0.75	0.80	75	56.25	531.1
	200	350	300	2.50	110.0	10.0	3.08	Hooked	1.00	2000	0.75	0.80	75	56.25	287.2
	200	350	300	3.50	111.5	10.0	3.08	Hooked	1.00	2000	0.75	0.80	75	56.25	211.6
	200	350	300	4.50	110.8	10.0	3.08	Hooked	1.00	2000	0.75	0.80	75	56.25	214.7
Huang et al.	150	300	255	1.96	55.8	20.0	4.93	Chopped with butt	0.50	700	1.00	0.66	47	23.33	254.0
Kwak et al.	152	305	283	2.50	33.1	9.5	1.99	Hooked	1.00	1100	1.00	0.51	100	100.00	136.6
	152	305	283	2.50	33.2	9.5	1.99	Hooked	1.00	1100	1.00	0.51	100	100.00	145.5
	152	305	283	2.50	33.0	9.5	1.99	Hooked	1.00	1100	2.00	0.51	100	200.00	134.4
	152	305	283	2.50	34.4	9.5	1.99	Hooked	1.00	1100	2.00	0.51	100	200.00	138.8
Roberts & Ho	50	200	170	2.41	32.1	10.0	2.37	Brass-coated steel	0.50	1100	3.00	0.38	100	150.00	32.9
	50	200	170	2.41	39.3	10.0	2.37	Brass-coated steel	0.50	1100	4.50	0.38	100	225.00	36.3
	50	200	170	1.62	32.1	10.0	2.37	Brass-coated steel	0.50	1100	3.00	0.38	100	150.00	51.0
	50	200	170	1.62	39.3	10.0	2.37	Brass-coated steel	0.50	1100	4.50	0.38	100	225.00	54.3

	50	200	170	0.81	32.1	10.0	2.37	Brass-coated steel	0.50	1100	3.00	0.38	100	150.00	81.2
	50	200	170	0.81	39.3	10.0	2.37	Brass-coated steel	0.50	1100	4.50	0.38	100	225.00	107.6
Hwang et al.	100	200	166	3.02	39.4	10.0	3.43	Hooked	1.00	1200	0.50	0.50	60	30.00	30.9
	100	200	166	3.02	39.2	10.0	3.43	Hooked	1.00	1200	1.00	0.50	60	60.00	52.4
	100	200	166	3.02	40.0	10.0	3.43	Hooked	1.00	1200	1.50	0.50	60	90.00	54.4
	100	200	166	3.02	35.5	10.0	3.43	Hooked	1.00	1200	2.00	0.50	60	120.00	47.9
	100	200	159	3.14	58.0	10.0	4.78	Hooked	1.00	1200	1.00	0.50	60	60.00	74.1
	100	200	159	3.14	80.1	10.0	4.78	Hooked	1.00	1200	0.50	0.50	60	30.00	73.1
	100	200	159	3.14	88.0	10.0	4.78	Hooked	1.00	1200	1.00	0.50	60	60.00	81.5
Spinella et al.	150	250	219	2.80	80.0	10.0	1.91	Hooked	1.00	1100	1.00	0.55	55	54.55	114.8
	150	250	219	2.00	80.0	10.0	1.91	Hooked	1.00	1100	1.00	0.55	55	54.55	141.9
Chalioris & Sfiri	100	300	275	2.00	28.4	9.5	0.55	Hooked	1.00	1100	0.50	0.80	75	37.50	43.0
Cohen & Aoude	125	250	212	3.77	59.4	10.0	1.52	Hooked	1.00	1100	0.50	0.55	55	27.27	44.3
Aoude & Cohen	125	250	212	3.77	49.6	10.0	1.52	Hooked	1.00	1100	0.50	0.38	80	40.00	45.9
	125	250	210	3.81	49.7	10.0	2.28	Hooked	1.00	1100	0.75	0.55	55	41.25	44.9
	125	250	210	3.81	51.5	10.0	2.28	Hooked	1.00	1100	1.00	0.55	55	55.00	58.9
	125	250	210	3.81	54.5	12.0	2.28	Hooked	1.00	1100	1.00	0.55	55	55.00	59.9
Qissab & Salman	100	170	140	1.07	36.1	12.5	1.12	Hooked	1.00	1100	0.50	0.80	63	31.26	73.4
	100	170	140	1.07	36.9	12.5	1.12	Hooked	1.00	1100	0.75	0.80	63	46.89	87.1
	100	170	140	2.50	36.1	12.5	1.12	Hooked	1.00	1100	0.50	0.80	63	31.26	40.8
	100	170	140	2.50	36.9	12.5	1.12	Hooked	1.00	1100	0.75	0.80	63	46.89	51.1
	100	180	150	1.00	36.1	12.5	1.05	Hooked	1.00	1100	0.50	0.80	63	31.26	106.7
	100	180	150	1.00	36.9	12.5	1.05	Hooked	1.00	1100	0.75	0.80	63	46.89	126.0
	100	180	150	2.33	36.1	12.5	1.05	Hooked	1.00	1100	0.50	0.80	63	31.26	44.7
	100	180	150	2.33	36.9	12.5	1.05	Hooked	1.00	1100	0.75	0.80	63	46.89	46.9
	100	200	170	2.41	36.1	12.5	0.92	Hooked	1.00	1100	0.50	0.80	63	31.26	42.3
	100	200	170	1.29	36.1	12.5	0.92	Hooked	1.00	1100	0.50	0.80	63	31.26	21.6
	100	275	245	0.90	36.1	12.5	0.64	Hooked	1.00	1100	0.50	0.80	63	31.26	51.4
Furlan & de Hanai	100	100	85	3.52	54.8	10.0	1.66	Crimped	0.75	1100	1.00	0.20	127	95.25	20.1
	100	100	85	3.52	50.0	10.0	1.66	Crimped	0.75	1100	2.00	0.20	127	190.50	22.1
	100	100	85	3.52	49.3	10.0	1.66	Crimped	0.75	1100	1.00	0.20	191	142.88	21.6
	100	100	85	3.52	49.3	10.0	1.66	Crimped	0.75	1100	1.00	0.20	191	142.88	18.6
	100	100	85	3.52	53.7	10.0	1.66	Crimped	0.75	1100	2.00	0.20	191	285.75	20.1

	100	100	85	3.52	53.5	10.0	1.66	Crimped	0.75	1100	0.50	0.20	191	71.44	22.6
	100	100	85	3.52	53.5	10.0	1.66	Crimped	0.75	1100	0.50	0.20	191	71.44	17.6
Danygier & Savir	200	325	273	2.75	110.9	22.0	3.48	Hooked	1.00	1000	0.75	0.55	64	47.73	202.2
	200	325	273	2.75	109.2	22.0	3.48	Hooked	1.00	1000	0.75	0.90	67	50.00	210.9
Krassowska & Kosior-Kazberuk	80	180	165	2.99	41.2	4.0	1.71	Hooked	1.00	800	1.00	1.00	50	50.00	32.9
	80	180	165	2.99	39.9	4.0	1.71	Hooked	1.00	800	1.50	1.00	50	75.00	41.4
Yoo & Yang	300	500	420	3.21	62.3	20.0	3.22	Hooked	1.00	1400	0.75	0.55	65	48.75	417.4
	450	750	648	3.26	62.3	20.0	3.27	Hooked	1.00	1400	0.75	0.55	65	48.75	815.0
	600	1000	887	3.26	62.3	20.0	3.43	Hooked	1.00	1400	0.75	0.55	65	48.75	1480.9
Gali & Subramaniam	125	250	222	1.80	30.0	10.0	1.45	Hooked	1.00	1225	0.50	0.75	80	40.00	79.5
	125	250	222	1.80	30.0	10.0	1.45	Hooked	1.00	1225	0.50	0.75	80	40.00	86.5
Zamanzadeh et al.	70	300	270	2.56	50.0	10.0	3.32	Recycled	0.75	1100	0.77	0.25	58	33.45	81.6
	110	300	270	2.56	50.0	10.0	2.12	Recycled	0.75	1100	0.77	0.25	58	33.45	96.2
	150	300	270	2.56	50.0	10.0	1.55	Recycled	0.75	1100	0.77	0.25	58	33.45	109.8
Shoaib et al.	310	308	258	3.00	23.0	10.0	2.50	Hooked	1.00	1100	1.00	0.55	55	54.55	211.8
	310	308	240	3.00	41.0	10.0	4.03	Hooked	1.00	1100	1.00	0.55	55	54.55	281.7
	310	308	258	3.00	41.0	10.0	2.50	Hooked	1.00	1100	1.00	0.55	55	54.55	278.8
	310	308	240	3.00	80.0	10.0	4.03	Hooked	1.00	1100	1.00	0.55	55	54.55	459.2
	300	600	531	3.00	23.0	10.0	1.88	Hooked	1.00	1100	1.00	0.55	55	54.55	255.2
	300	600	523	3.00	23.0	10.0	2.55	Hooked	1.00	1100	1.00	0.55	55	54.55	245.1
	300	600	523	3.00	41.0	10.0	2.55	Hooked	1.00	1100	1.00	0.55	55	54.55	447.1
	300	1000	923	3.00	41.0	10.0	1.44	Hooked	1.00	1100	1.00	0.55	55	54.55	499.8
	300	1000	920	3.00	41.0	10.0	2.03	Hooked	1.00	1100	1.00	0.55	55	54.55	504.7
	300	1000	923	3.00	80.0	10.0	1.44	Hooked	1.00	1100	1.00	0.55	55	54.55	653.3
	300	1000	920	3.00	80.0	10.0	2.03	Hooked	1.00	1100	1.00	0.55	55	54.55	651.2
Bae et al.	200	350	300	3.50	215.0	2.0	3.60	Hooked	1.00	1100	2.00	0.55	55	109.09	373.5
	200	350	300	2.00	199.0	2.0	3.60	Hooked	1.00	1100	2.00	0.55	55	109.09	586.6
Abdul-Zaher et al.	120	300	266	1.13	31.9	20.0	1.26	Corrugated	0.75	834	0.20	1.00	50	7.50	126.7
	120	300	266	1.13	31.9	20.0	1.26	Corrugated	0.75	834	0.40	1.00	50	15.00	133.2
	120	300	266	1.13	31.9	20.0	1.26	Corrugated	0.75	834	0.60	1.00	50	22.50	146.1
